# Entropic Mixing Allows Monomeric‐Like Absorption in Neat BODIPY Films

**DOI:** 10.1002/chem.202002463

**Published:** 2020-10-01

**Authors:** Clara Schäfer, Jürgen Mony, Thomas Olsson, Karl Börjesson

**Affiliations:** ^1^ Department of Chemistry and Molecular Biology University of Gothenburg Kemigården 4 412 96 Gothenburg Sweden

**Keywords:** BODIPY, chromophores, entropic mixing, morphology, strong exciton-photon coupling

## Abstract

Intermolecular interactions play a crucial role in materials chemistry because they govern thin film morphology. The photophysical properties of films of organic dyes are highly sensitive to the local environment, and a considerable effort has therefore been dedicated to engineering the morphology of organic thin films. Solubilizing side chains can successfully spatially separate chromophores, reducing detrimental intermolecular interactions. However, this strategy is also significantly decreasing achievable dye concentration. Here, five BODIPY derivatives containing small alkyl chains in the α‐position were synthesized and photophysically characterized. By blending two or more derivatives, the increase in entropy reduces aggregation and therefore produces films with extreme dye concentration and, at the same time almost solution like absorption properties. Such a film was placed inside an optical cavity and the achieved system was demonstrated to reach the strong exciton‐photon coupling regime by virtue of the achieved dye concentration and sharp absorption features of the film.

## Graphical abstract

**Entropic mixing** of two or more, highly similar dye molecules results in thin films showing almost solution like photophysical properties. Mixed films of new, low molecular weight BODIPY dyes, with short alkyl chains, were fabricated and furthermore used in an optical cavity, where the strong coupling regime was reached.

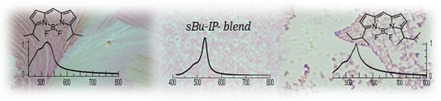

Organic dyes have a high tendency to aggregate due to their flat structure and large polarizability. The close dye–dye proximity give rise to strong interchromophore interactions resulting in new and often unwanted effects such as H‐ and J‐aggregates,^[1]^ excimers,^[2]^ charge transfer states,^[3]^ and traps for charges.^[4]^ In materials chemistry, molecules ability to aggregate plays a crucial role and a considerable effort has therefore been dedicated to engineering the morphology of organic thin films.^[5]^ Self‐assembly of chromophores is highly dependent on interactions between the single molecules, especially π–π interactions between the chromophore cores π‐unit. Strong intermolecular interactions are extremely sensitive on relative distances and orientations, and the most widely adopted method of reducing these interactions are by adding large bulky substituents to the chromophore core.^[6]^ These substituents, whose van der Waal interactions are known to interplay with the π–π interactions of the chromophore cores, enclose the chromophores, insulating it from the environment, and thereby enable unperturbed photophysical properties in neat films.[^6b^, ^7^] However, substituents are often much larger than the chromophore itself, causing an effective concentration decrease, limiting the method to applications where the concentration and intermolecular communication is less important.^[8]^

Strong light‐matter coupling using organic molecules is a relatively new research field, but has already harvested considerable attention.^[9]^ Landmarks such as selective chemical reactions,^[10]^ room temperature Bose–Einstein condensation,^[11]^ and modification of singlet and triplet energy levels,^[12]^ has for instance been achieved in the strong coupling regime. The strength of the exciton‐photon coupling depends on the ability of a molecular film to absorb light. The coupling strength is proportional to the transition dipole moment of the coupled transition and the square root of the concentration of molecules. Furthermore, the absorption envelope should be sharp as to minimize dissipation of energy from the system. Highly absorbing and small molecules having the ability of being processed into neat films are therefore a limiting factor within this research field.

Being half a porphyrin in size, BODIPY (boron dipyrromethene) dyes have been shown to have a broad spectrum of applications. Their excellent photophysical properties such as high fluorescence quantum yields, large molar absorptivity, narrow absorption and emission bands, small Stokes shifts, tuneable emission, and a high photo‐ and chemical stability make them highly versatile.^[13]^ They are used as biological labels,^[14]^ as fluorescent switches,^[15]^ photosensitizers^[16]^ and as laser‐dyes,^[17]^ to just name a few of their applications. Although BODIPY dyes perform excellent when in solution, in neat films, their main absorption band broadens considerable and emission yields diminish, limiting their application range. The cause for this are strong intermolecular interactions between the π‐core of the molecule which leads to aggregation and thus scattering as well as highly perturbed photophysical properties.^[18]^ BODIPY dyes in the strong coupling regime have been studied by the group of Lidzey. They used BODIPY derivatives doped at 10–20 % in an optically inert polymer matrix to avoid aggregation, and by doing so explored the optical properties^[19]^ and the energy transfer between two derivatives.^[9b]^ Furthermore, polariton lasing,^[20]^ formation of polariton condensates^[21]^ and anti‐stokes fluorescence^[22]^ have been successfully shown.

Entropic mixing uses entropy as a mean to reduce a systems ability to aggregate.^[23]^ The method utilizes the entropy increase of blends of photophysically indistinguishable but physically different chromophores to reduce the Gibbs free energy of the amorphous state.^[24]^ This effectively counteracts intermolecular interaction and therefore reduces the system's ability to aggregate. In theory, the method therefore offers the possibility to make thin organic films having very high molecular concentrations but still monomeric like photophysical properties.

In this work, five BODIPY dyes with linear as well as branched alkyl chains in the α‐position were synthesized with the discovery of a previously unknown substitution effect that enabled a fourfold increase in yield of the boron complexation. The photophysical properties of the BODIPY dyes in solution were assessed to be indistinguishable using steady state and time resolved optical spectroscopy. Neat films showed a high tendency to aggregate, considerable light scattering, and the sharp BODIPY absorption features were considerable broadened. Films consisting of mixed derivatives having branched substituents showed reduced light scattering and monomeric like absorption features. However, when derivatives having branched and linear substituents were mixed together, phase separation became evident, highlighting the importance of using very similar substituents in entropic mixing schemes. Finally, to demonstrate on the applicability of the concept, a film consisting of mixed BODIPY derivatives where encapsulated within an optical cavity, and the attained system was determined to reach the strong coupling regime, due to the strong and narrow absorption band of the BODIPY film.

BODIPY is a strongly absorbing dye in solution, but in the solid state, a variety of aggregates result in strong interchromophore interactions and hence a change in the photophysical properties. To enable highly concentrated BODIPY films with monomeric like photophysical properties, the method of entropic mixing was employed. The synthesis of five BODIPY derivatives, having ethyl (Et‐BODIPY), *n*‐butyl (*n*Bu‐BODIPY), iso‐propyl (IP‐BODIPY), *sec*‐butyl (*s*Bu‐BODIPY) and *tert*‐butyl (*t*Bu‐BODIPY) substituents was successfully conducted via alkylation of pyrrole, followed by condensation and complexation (Scheme 1).

**Scheme 1:**
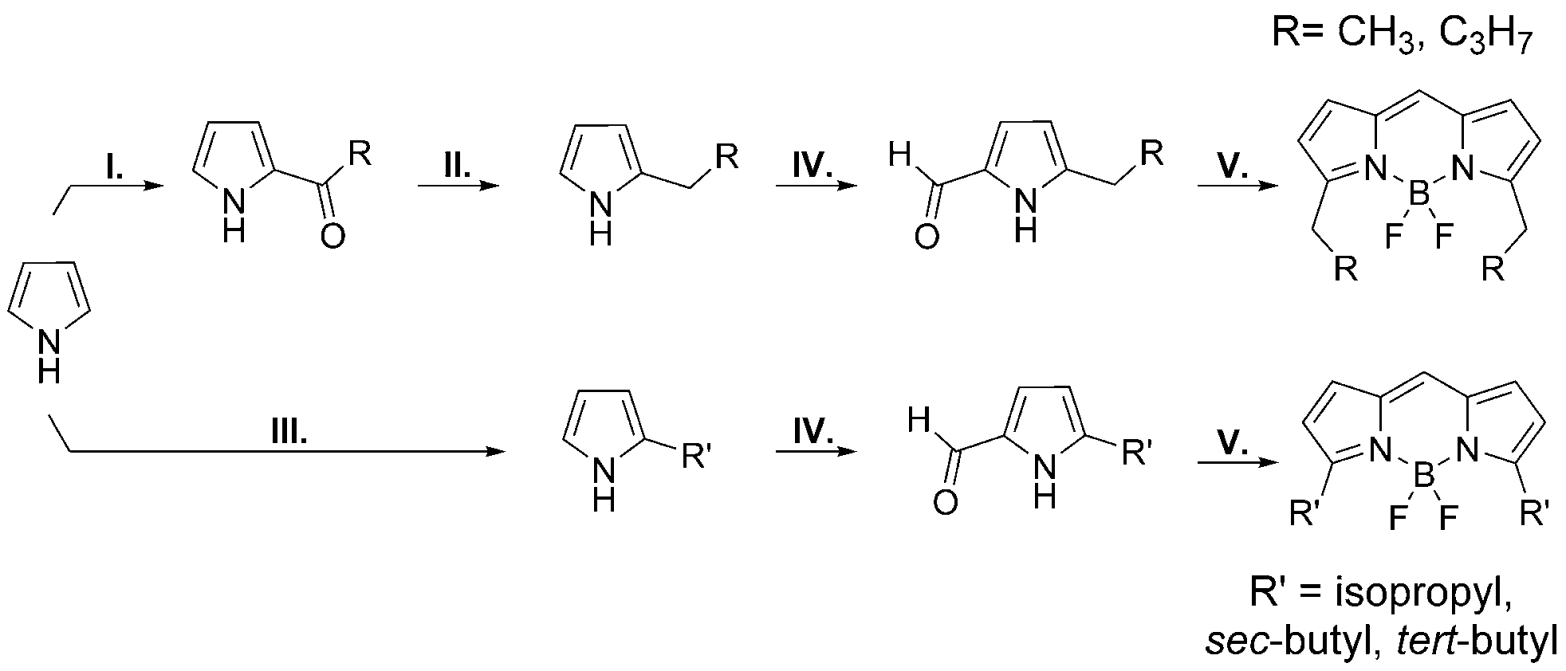
Synthesis of α‐alkyl BODIPY dyes. I.) 1. POCl_3_, (CH_3_)_2_NOOR, DCE; 2. NaOAc, H_2_O. II.) LiAlH_4_, THF or NaBH_4_, IPA. III.) 1. MgI, Et_2_O; 2. BrR′, Et_2_O 3. NH_4_Cl_aq_, IV.) 1. POCl_3_, DMF, DCE; 2. NaOAc, H_2_O. V.) 1. POCl_3_, DCM; 2. Et_3_N, BF_3_⋅OEt_2_.

Alkylation of pyrrole with linear sidechains was achieved by performing a Vielsmeier‐Haack type reaction using *N*,*N*‐dimethylalkylamides^[25]^ and POCl_3_ to form the corresponding pyrrylketones, and a following reduction of the carbonyl moiety to the alkane, using either LiAlH_4_ or NaBH_4_. The alkylated pyrroles were subsequently submitted to a Vielsmeier‐Haack formylation, using DMF and POCl_3_, which afforded the 2‐alkyl‐5‐formylpyrroles that were used for the BODIPY‐condensation.^[26]^ Substitution of branched alkyl chains was accomplished by a Grignard reaction via the formation of a pyrrolegrignard, which was treated with the designated alkyl bromide to afford a mix of α‐ and β‐substituted alkyl pyrroles.^[27]^ As the formed pyrroles could not be separated properly via distillation or column chromatography, the regioisomer mixtures were subjected to the next step, a Vielsmeier‐Haack formylation, and the obtained 2‐ and 3‐alkyl‐5‐formylpyrroles could be separated using column chromatography. The prepared 2‐alkyl‐5‐formylpyrroles were treated with POCl_3_ to undergo condensation to the dipyrromethene‐intermediate, which were directly treated with Et_3_N and BF_3_⋅OEt_2_ to perform the complexation to the desired BODIPY dyes.

These last two steps produced yields in the range of 10 to 50 %. The compounds with linear sidechains only gave yields of ∼10 %, whereas adding a small branched sidechain like isopropyl increased the yields substantially. The α‐isopropyl‐pyrrole yielded 44 % and the α‐*sec*butyl‐pyrrole yielded 49 % of the desired product. Adding a more bulky sidechain though, like a *tert*‐butyl group, only gave the product in 15 % yield, which agrees well with earlier reported values.^[27c]^ This leads to the conclusion that steric bulk in the α‐position either prevent side reactions or increase the stability of the dipyrromethene‐intermediate (based on the proposed mechanism of this reaction, there is no reason why steric bulk should affect the rate of the reaction^[8]^). However, adding a too bulky substituent seems to create a steric hindrance, most probably in the complexation step, lowering the reaction yield. In summary, five BODIPY derivatives with ethyl, isopropyl, *n*‐butyl, *sec*‐butyl or *tert*‐butyl substituents were synthesised, with the surprising discovery that the right amount of bulkiness on the α‐position significantly increases the yield of the complexation reaction.

Entropic mixing requires the blended molecules to exhibit indistinguishable photophysical properties.^[24]^ To verify that the prepared BODIPY derivatives satisfy this criterion, steady‐state and time resolved optical spectroscopy was performed. The five synthesized alkylated derivatives display almost identical absorption and emission envelopes when in solution (Figure 1). Et‐, *n*Bu‐, *s*Bu‐ and IP‐BODIPY show sharp absorption and emission bands as well as very small Stokes shifts, large fluorescence quantum yields, single exponential excited state lifetimes and high molar absorptivities (Table 1, Figure S2). However, *t*Bu‐BODIPY has a slightly broader absorption and emission profile as well as a larger stokes shift as compared to the others. This difference in the photophysical behaviour is probably due to a distortion of the fluorines, caused by the sterically demanding *tert*‐butyl groups at the α‐positions of the BODIPY core. In conclusion, the photophysical properties of the prepared BODIPY‐derivatives are indistinguishable from each other (perhaps with the exception of *t*Bu‐BODIPY). The BODIPY‐derivatives can therefore be mixed together in order to achieve high entropy blends with monomeric like photophysical properties.

**Figure 1:**
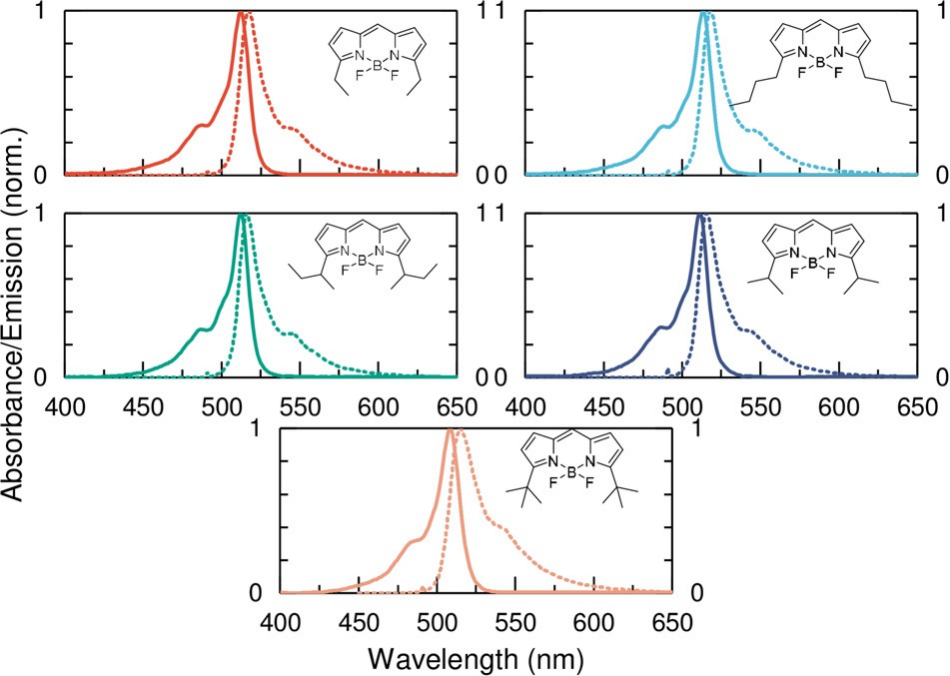
Absorption (solid line) and emission (dotted line) of the BODIPY derivatives in DCM solution.

**Table 1:** Photophysical data of BODIPY‐derivatives in DCM solution.

	Et	*n*Bu	*s*Bu	IP	*t*Bu
*λ*_max abs_ [nm]	512	513	512	511	509
*λ*_max em_ [nm]	517	518	516	515	515
Stokes shift [cm^−1^]	189	188	151	152	229
*Φ*_f_	0.90	0.85	1.0	0.93	1.0
*τ* [ns]	5.42	5.26	5.09	5.15	5.35
*ϵ* [M^−1^ cm^−1^]	97 000	98 000	98 000	101 000	100 000

The BODIPY core is flat, providing the possibility for molecular stacking. Furthermore, its low energy high intensity electronic transition indicates a large polarizability, and thus large London dispersive forces, further reinforcing the molecules ability to aggregate. The physical and photophysical properties of pristine films of the BODIPY‐derivatives were first studied, and these were later compared to those of blended systems. All five derivatives produce pristine films having a significant degree of aggregated domains as revealed by polarized microscopy (Figures S3 and S4). Bulky‐ and linear substituents all seem to give rise to similar aggregation, although *n*Bu shows the largest aggregated domain sizes, and IP more rod like aggregates than the others. When chromophores are tightly packed together, interactions between their transition dipole moments can be so strong that the energetics are perturbed. The best‐known examples of intermolecular strong coupling is H‐ and J‐aggregates.^[1]^ All pristine films of the derivatives scatter light to a large degree and show broad absorption bands (Figure S5). This is quite unlike the sharp transitions in dilute solution, and it indicates an inhomogeneous environment for the molecules in the films. An inhomogeneous environment is also evident when observing emission from the films. All exhibit broad and structured emission, although two very distinct emission bands are clear in *s*Bu and IP films, indicating at least two distinct type of aggregates. The emission lifetime of these two emissive states differs substantially, where the low‐energy transition always is the long‐lived one (Figure S6). To summarize, pristine films of the BODIPY derivatives have a substantial degree of aggregation causing light scattering and a highly broadened absorption profile.

Pristine films of the derivatives display a broad absorption profile as well as significant light scattering. These undesirable features are due to aggregated domains, being large enough to scatter light, and providing a close packing that results in strong interactions between the transition dipole moments of the individual chromophores. To see if entropy can be used as a means of disrupt packaging without diluting the concentration compared to pristine films, the derivatives were blended together. The properties of blended films can be classified into two categories, those consisting of binary blends of IP, *s*Bu, and *t*Bu that do not show any large aggregation domains, and others that show signs of large aggregates forming (Figures S7–S10). In blends containing linear alkyl chain derivatives, rod like aggregate structures were observed, leading to broad absorption and emission bands. Figure 2 displays absorption and emission spectra of selected blended films (see Figure S11 for other blend combinations). For the IP‐*s*Bu blend, very little scattering is present and the linewidth of the main absorption band is considerable narrower as compared to pristine films. The absorption maximum is at 532 nm, a small redshift as compared to the solution value of 512 nm. The emission shows two distinct bands, one sharp with a small Stokes shift, in shape resembling the emission from dilute solution, and another redshifted and slightly broader. Other combinations containing *s*Bu show similar photophysical properties (as for the *s*Bu‐IP blend). To assess the effect of blending on the emission quantum yield, the quantum efficiency of the *s*Bu‐IP blend as well as the individual components, *s*Bu and IP, were determined to 15, 8, and 2 %, respectively. This can be compared to earlier reports of quantum efficiencies in the range of 7–13 % for BODIPYs doped at 15 w/w% in polymers.^[28]^ Blending thus outperforms single components and allows for much higher concentrations compared to dyes dispersed in polymers with retained quantum efficiencies.

**Figure 2:**
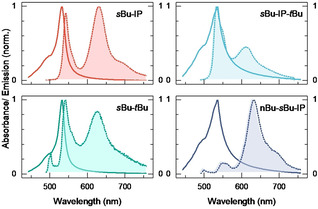
Absorbance and emission of a selection of blended films (see Figure S11 for more combinations). The mixed films were prepared from toluene solutions of the BODIPY dyes (*c*_dye_=0.003 m). The ratio of the components used in the mixes is 1:1 and 1:1:1 respectively.

To examine if the low energy emission band is the result from excimeric or J‐aggregate like states, time resolved emission spectroscopy was performed. The decay of emission from the *s*Bu‐*t*Bu blend film was recorded at the maximum of the two emission bands (Figure S14 and S15). The average lifetime of the high energy monomeric like emission band is 1.0 ns, and the average lifetime of the low energy emission band is 3.3 ns. A global analysis of the decays from the two emission bands reveals a build‐up of low energy emission with the same time constant as the high‐energy emission decay. This indicates that these two emissive states do not come from different locations in the film, but rather that throughout the film the low energy state acts as an energy relaxing route from the high energy monomeric excited state. The absence of strong absorption in the wavelength range of the low energy emission indicates that either the aggregational state is present in low concentrations, or it has a low transition dipole moment. Furthermore, absorbance of the low energy transition is not seen in excitation spectra (Figure S12). The relative long lifetime of the low energy state supports excimer formation, whereas the relative narrow bandwidth of the emission advocates J‐aggregates. Previous studies have pointed to the existence of J‐aggregated dimers in BODIPY‐polymer mixes.^[28]^ Other combinations of mixtures show broader absorption features, indicating that the type of aggregates present in the various films might differ.

Interestingly, we see emission around 500 nm in various blends and in neat IP films. Considering the large emission quantum yield, and that the feature is present in different blend combinations, we assign this feature to an aggregational state rather than an impurity. Excitation spectroscopy reveals that the state being responsible for the emission only absorbs on the high energy shoulder of the film (Figure S12). In summary, the absorbance features from thin films consisting of blends of *s*Bu and IP/*t*Bu is sharp and monomeric like. This indicates that for entropic mixing to be successful, very similar substituents should be used as to reduce possible phase segregation. The blending furthermore reduces light scattering due to a reduced amount of large aggregates.

To reach the strong light‐matter coupling regime, strongly absorbing dyes need to be processed at high concentrations in an optical cavity. The optical cavity provides a mean to enhance the electromagnetic field strength at the frequency of the molecular electronic transition. This by having a cavity thickness that supports a standing wave with the same energy as the BODIPY absorption. Now energy is interchanged between the cavity and the dye faster than energy dissipation, and the strong coupling regime is reached. When this happens, the molecular transition is replaced by two polaritonic states, the upper and lower polaritons, located at higher and lower energy as compared to the molecular transition, respectively (Figure 3 a). The polaritonic states consist of contributions from both light and matter, and have therefore exotic properties such as being delocalized over the entire cavity. Figure 3 b displays angular resolved reflectivity of a Fabry–Pérot cavity containing a BODIPY film (*s*Bu‐IP 1:1). The BODIPY film is sandwiched between layers of polyvinyl alcohol (Figure S1), which insulates the film from the Ag mirrors in order to remove possible disturbances from Ag surface plasmons. The molecular transition is clearly replaced by two polaritonic states. As the angle of reflectivity increases, the energy of the lower polariton approaches the energy of the bare molecule transition, but never cross it. The observed avoided crossing is a typical feature of a strongly coupled system. The Rabi splitting (the minimal splitting between the upper and lower polariton) was calculated to 358 meV by fitting the angular resolved reflectivity to a coupled oscillators’ model (see methods section). This value is considerably larger than the full width at half maximum of the molecular absorbance (115 meV) of the film, and the condition for entering the strong coupling regime is therefore met. Thus, the ability to process BODIPY in small films at extreme concentrations (about 3.1 m, assuming a density of 1.0 g cm^−3^) without a significant broadening of the absorption envelope, enables the strong exciton‐photon coupling regime to be reached.

**Figure 3:**
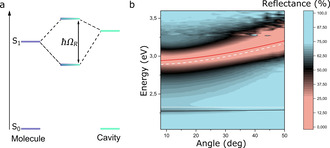
a) Energy diagram showing how the molecular transition couples to a cavity mode to form the two polaritonic states, the upper (UP) and lower (LP) polaritons. b) Angle resolved reflectivity of the optical cavity, red and black line show a fit to a coupled oscillator model, which as used to extract the coupling strength, where the black line shows the lower polariton, the red line the upper polariton, the white dashed line the energy of the cavity mode, and the white dotted line the transition energy of the molecule.

In conclusion, five different BODIPY derivatives, alkylated in the α‐position, were synthesized. The highest achieved yield in the boron complexation step was 49 %, which is considerable higher than previously reported yields for this kind of compounds. This is presumably due to a certain steric bulk of the introduced alkyl sidechains, although support for this assumption cannot be rationalized by the suggested mechanism of BODIPY condensation using POCl_3_.^[26]^ As expected, all five derivatives show very similar photophysical properties when in solution. Unlike the properties of the dyes in solution, neat films of the BODIPY derivatives show broad absorption and emission bands. Blending of two or more derivatives leads to a notable improvement of thin film properties. Some blends exhibit almost solution like absorption and emission features. Furthermore, the increased entropy in mixed films reduces the molecules ability to aggregate, which is observed as a reduction in light scattering due to a lower amount of large aggregated domains. To demonstrate on the importance of thin films having sharp absorption features and extreme dye concentrations, a blended film was placed in an optical cavity, and the achieved system reached the strong coupling regime. Given the prospects shown here, we envision that entropic mixing will become a standard tool as to control thin film morphology within materials science.

## Conflict of interest

The authors declare no conflict of interest.

## Supporting information

As a service to our authors and readers, this journal provides supporting information supplied by the authors. Such materials are peer reviewed and may be re‐organized for online delivery, but are not copy‐edited or typeset. Technical support issues arising from supporting information (other than missing files) should be addressed to the authors.

Supplementary
