# Supplementary material for: Entropic Mixing Allows Monomeric‐Like Absorption in Neat BODIPY Films
Source: Chemistry. 2020 Oct 1;26(63):14295–9. doi: 10.1002/chem.202002463 (PMC7702096; doi:10.1002/chem.202002463)
Supplement: Supplementary file 1 — Supplementary [file CHEM-26-14295-s001.pdf]

# Chemistry–A European Journal

Supporting Information


## Entropic Mixing Allows Monomeric-Like Absorption in Neat BODIPY Films


Clara Schäfer, Jürgen Mony, Thomas Olsson, and Karl Börjesson*[a]

## Table of Contents

Experimental Procedures ..... 3
Methods and Materials ..... 3
Supplementary figures ..... 5
Synthesis ..... 13
2-Acetylpyrrole ..... 13
2-Ethylpyrrole ..... 13
*N,N*-Dimethylbutyramide ..... 13
1-(1H-Pyrrol-2-yl)butan-1-one ..... 14
2-Butylpyrrole ..... 14
General Procedure A: to make the branched alkylated pyrroles a modified literature procedure was used [6] ..... 14
2-*sec*Butylpyrrole ..... 14
2-Isopropylpyrrole ..... 15
2-*tert*Butylpyrrole ..... 15
2-Butyl-5-formylpyrrole ..... 15
General Procedure B: for the Vielsmeier-Haack Formylation a literature procedure was used [8] ..... 15
2-Ethyl-5-formylpyrrole ..... 16
2-Isopropyl-5-formylpyrrole ..... 16
2-*sec*Butyl -5-formylpyrrole ..... 16
2-*tert*Butyl-5-formylpyrrole ..... 16
α-EthylBODIPY ..... 17
α-*tert*ButylBODIPY ..... 17
General Procedure C: for the BODIPY condensation ..... 17
α-ButylBODIPY ..... 18
α-*sec*ButylBODIPY ..... 18
α-IsopropylBODIPY ..... 18
NMR spectra ..... 19
2-Acetylpyrrole ..... 19
2-Ethylpyrrole ..... 19
*N,N*-Dimethylbutyramide ..... 20
1-(1H-Pyrrol-2-yl)butan-1-one ..... 20
2-Butylpyrrole ..... 21
2-Butyl-5-formylpyrrole ..... 22
2-Ethyl-5-formylpyrrole ..... 22
2-Isopropyl-5-formylpyrrole ..... 23
2-*sec*Butyl-5-formylpyrrole ..... 24
2-*tert*Butyl-5-formylpyrrole ..... 25
α-EthylBODIPY ..... 26
α-*tert*ButylBODIPY ..... 27
α-ButylBODIPY ..... 28
α-*sec*ButylBODIPY ..... 29
α-IsopropylBODIPY ..... 30
References ..... 31
Author Contributions ..... 31

## Experimental Procedures

### Methods and Materials

All reactions were carried out under nitrogen atmosphere unless stated differently. Glassware were oven dried prior to use. Unless indicated otherwise, common reagents, solvents, or materials were obtained from Sigma-Aldrich Chemical Co. and used without further purification. Dry solvents for reactions sensitive to moisture and/or oxygen were obtained through a solvent purifying system (MBRAUN SPS-800). Column chromatography was performed using silica gel (VWR 40 to 63 µm) unless stated otherwise. Flash chromatography was performed by a Teledyne CombiFlash EZ prep using normal-phase silica with a mesh size of 230 to 400, a particle size of 40 to 63 µm, and a pore size of 60 Å.
$^{1}H$ ($^{13}C$) NMR spectra were recorded on a Varian 400 spectrometer (400 MHz $^{1}H$; 100 MHz $^{13}C$) at room temperature using $CDCl_3$ (containing tetramethylsilane with 0.00 ppm as an internal reference) as solvent. Coupling constants (*J* values) are given in Hertz (Hz) and chemical shifts are reported in parts per million (ppm). High-resolution MS was obtained from an Agilent 1290 infinity LC system equipped with an auto sampler in tandem with an Agilent 6520 Accurate Mass Q-TOF LC/MS. Melting points were measured using a BÜCHI Melting Point B-545 instrument. IR spectra were recorded using an INVENIO R instrument from BRUKER.

**Optical microscopy**
Transmission optical micrographs were recorded with a Zeiss Axioscope 5 equipped with a pair of crossed polarizers.

**Sample preparation**
Neat films and cavities were prepared on glass substrates (25 × 25 mm), which were precleaned by sonication for 15 min in alkaline solution (0.5% of Hellmanex in distilled water), then rinsed with water and sonicated for 1 h in water and ethanol, respectively. The cleaned glass substrates were dried in an oven overnight prior to use. To avoid any inner filter effects, which are dependent on the thickness of the films, and to have absorbances below 0.1, the neat films as well as the blends were prepared to be very thin (oder of a few $10^{th}$ of nm). For neat films, solutions of the BODIPY dyes ($c_{dye}$ = 1.0 mg $mL^{-1}$) in toluene were spin coated (45 sec, R.T., 1500 rpm) (Laurell) on glass substrates. The ratio of the components used in the mixed films is 1:1, 1:1:1 and 1:1:1:1:1 respectively.
For the cavity, poly(vinyl alcohol) (PVA, 99+% hydrolyzed, Sigma Aldrich, 20 mg $mL^{-1}$) was dissolved in water and equal amounts of sBu- and IP BODIPY (each 2.25 mg $mL^{-1}$) were dissolved in a toluene solution of poly(2-vinylnaphtalene) (Sigma Aldrich, 0.5 mg $mL^{-1}$) resulting in a mass ratio of BODIPY dyes to polymer of 9:1. The layered structure of PVA, BODIPY and PVA on a silver mirror (120 nm) was made by step wise spin coating the corresponding solution on top of each other (45 sec, R.T., 1200 rpm). The total thickness of the three layers is roughly 115 nm. The optical cavity was sealed by depositing a second silver mirror (20 nm). The silver mirrors were fabricated by vacuum sputtering deposition (HEX, Korvus Technologies). The photonic contribution to the lower polariton is 8% whereas the excitonic contribution is 92%, at incident light beam. The contributions to the upper polariton are vice versa.

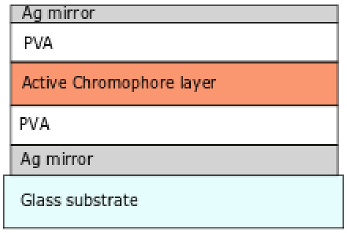

**Figure S1.** Cavity structure.

**Optical spectroscopy**
Reflectance spectra were measured using a spectrophotometer (LAMBDA 950, PerkinElmer) with a universal reflectance accessory. Steady-state emission spectra, excitation spectra and emission lifetimes were measured with a spectrofluorometer (FLS1000, Edinburgh Instrument). For the emission lifetime measurements, the samples were excited by a 475 nm picosecond pulsed diode laser (Edinburgh Instruments). The emission quantum yield were measured using the spectrofluorometer (FLS1000, Edinburgh Instrument) equipped with an integration sphere. As a reference a blank glass substrate was used.

SUPPORTING INFORMATION

**Coupled harmonic oscillator model**

The Rabi splitting was extracted by fitting the experimental data to the coupled harmonic oscillator model[1]. In the model, the coupling between one exciton and one photon is described by a $2 \times 2$ matrix Hamiltonian:

$$\begin{pmatrix} E_C(\theta) & \frac{\hbar\Omega_R}{2} \\ \frac{\hbar\Omega_R}{2} & E_X \end{pmatrix} \begin{pmatrix} \alpha \\ \beta \end{pmatrix} = E \begin{pmatrix} \alpha \\ \beta \end{pmatrix} \quad (1)$$

Where $E_X$ is the fixed exciton energy, $\hbar\Omega_R$ is the Rabi splitting, $\alpha$, $\beta$ are the mixing coefficients for the system (Hopfield coefficients) and $E_C(\theta)$ is the cavity energy, whose angle dependence follows equation (2).

$$E_C(\theta) = E_0 \left(1 - \frac{\sin^2\theta}{n_{eff}^2}\right)^{-\frac{1}{2}} \quad (2)$$

Where $E_0$ is the cavity energy at normal incidence, $\theta$ is the incidence and $n_{eff}$ is the effective refractive index.

## Supplementary figures

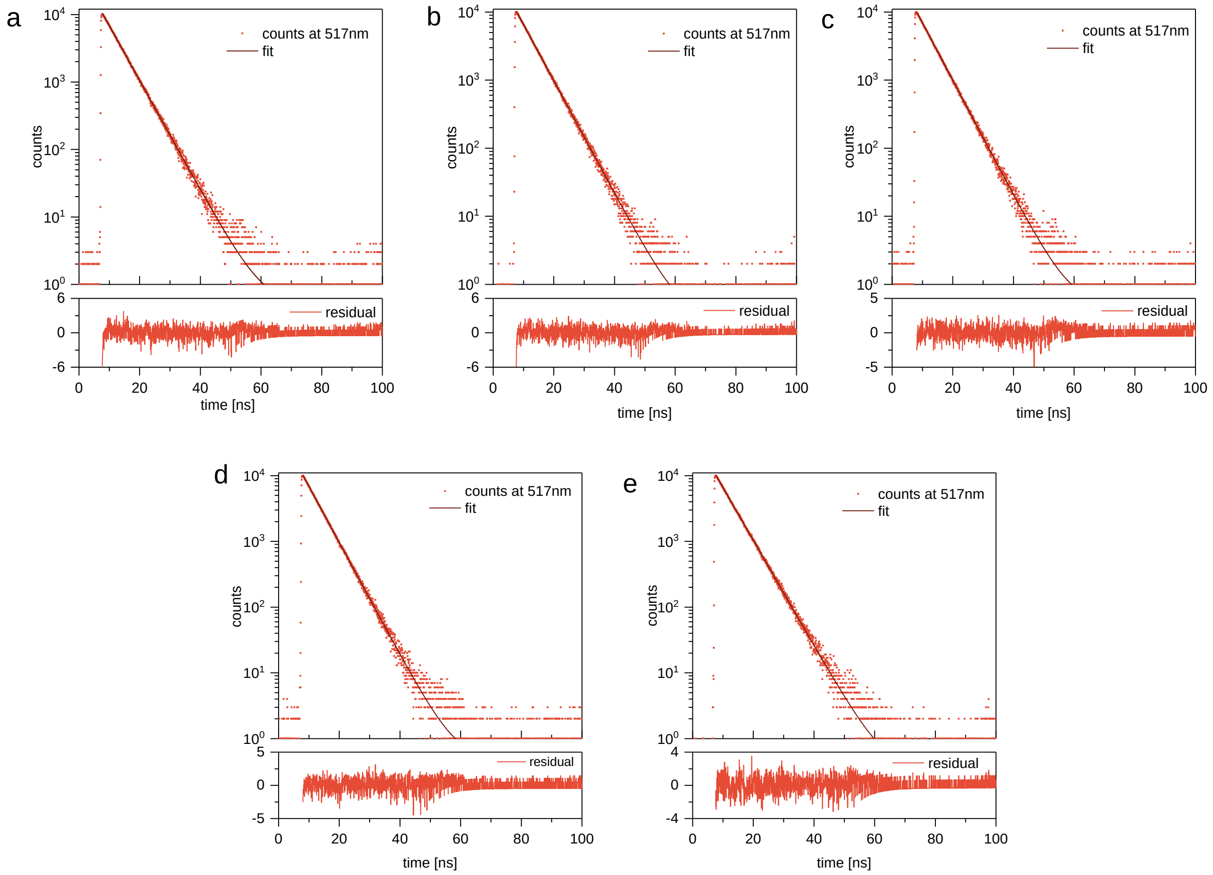


**Figure S2.** Emission decay of *α*-*tert*Butyl-BODIPY (a), *α*-nButyl-BODIPY (b), *α*-isopropyl-BODIPY (c), *α*-*sec*Butyl-BODIPY (d), and *α*-Ethyl-BODIPY (e) in DCM solution at 517 nm with the according fittings. The wavelength of excitation was 475 nm and 2048 channels were used when recording the decay.

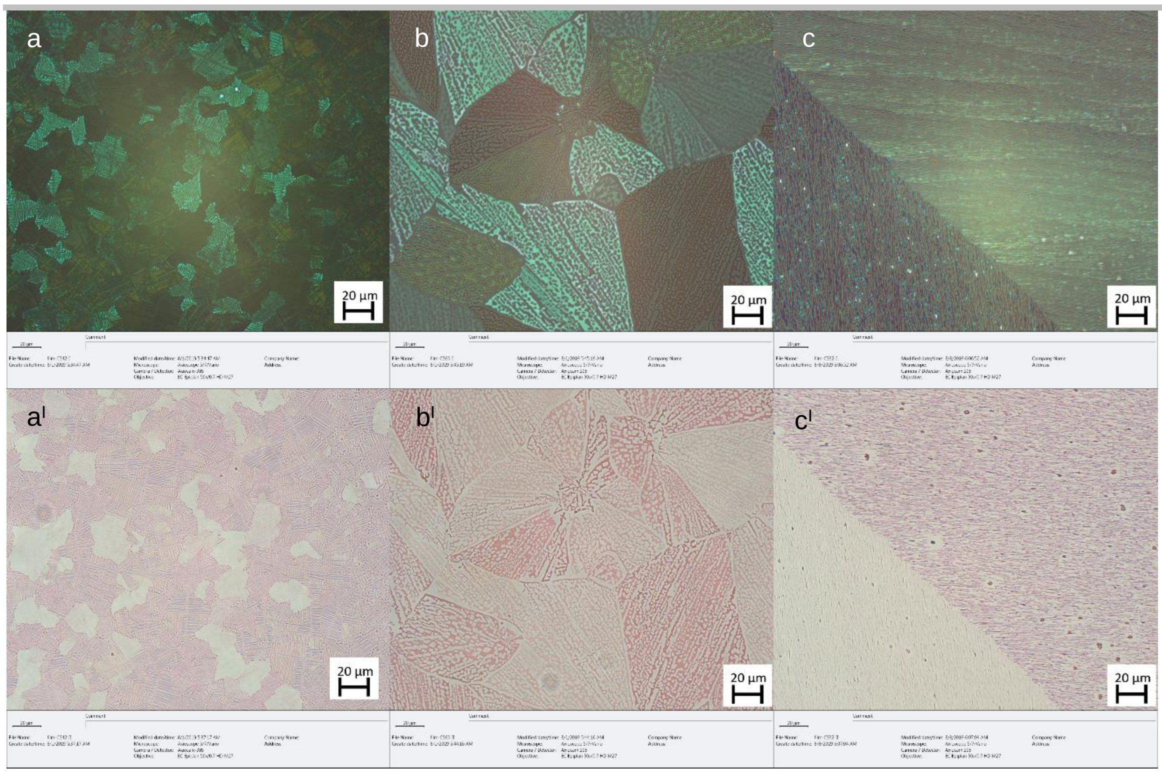


**Figure S3.** Transmission optical micrographs of neat BODIPY films of *s*Bu-BODIPY (a, $a^{I}$), *t*Bu-BODIPY (b, $b^{I}$) and nBu-BODIPY (c, $c^{I}$) with different directions of polarizers (top and bottom).

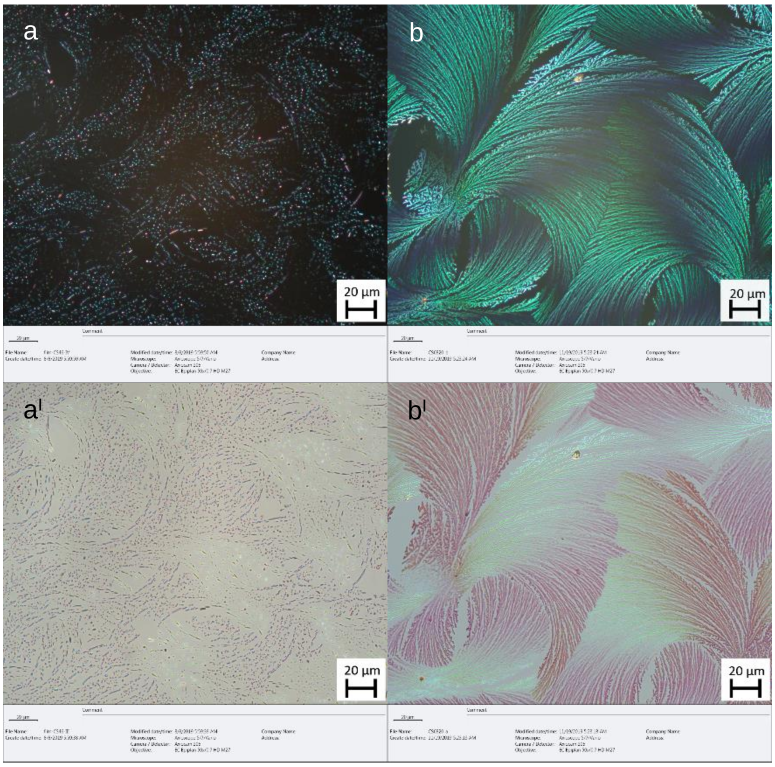


**Figure S4.** Transmission optical micrographs of neat BODIPY films Et-BODIPY (a, $a^{I}$) and IP-BODIPY (b, $b^{I}$) with different directions of polarizers (top and bottom).

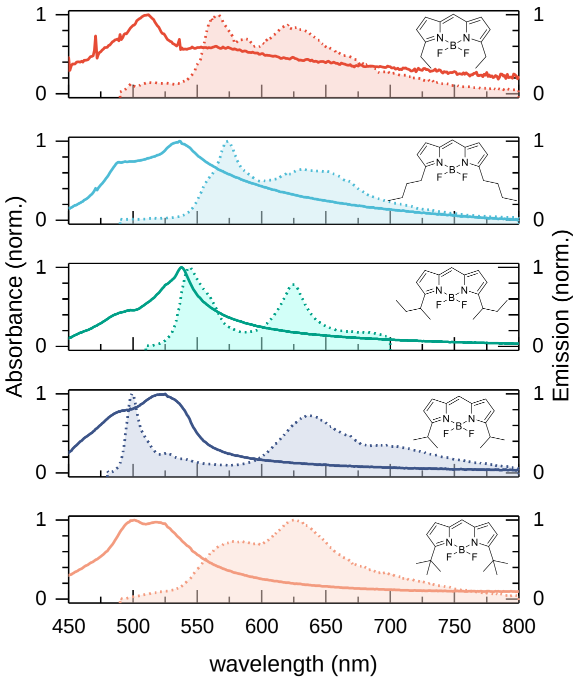


**Figure S5.** Absorbance and emission of pristine films.

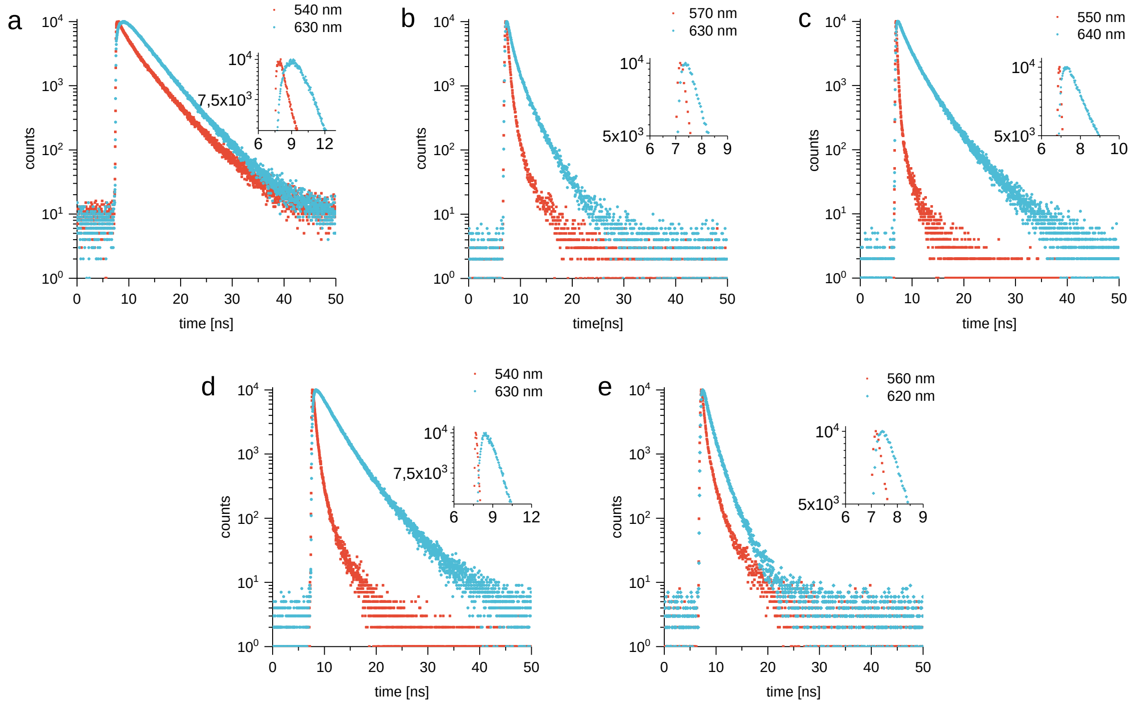


**Figure S6.** Emission decay of *α-tert*Butyl-BODIPY (a), *α*-nButyl-BODIPY (b), *α*-isopropyl-BODIPY (c), *α-sec*Butyl-BODIPY (d) and *α*-Ethyl-BODIPY (e) as pristine films. The wavelength of excitation was 475 nm and 2048 channels were used when recording the decay.

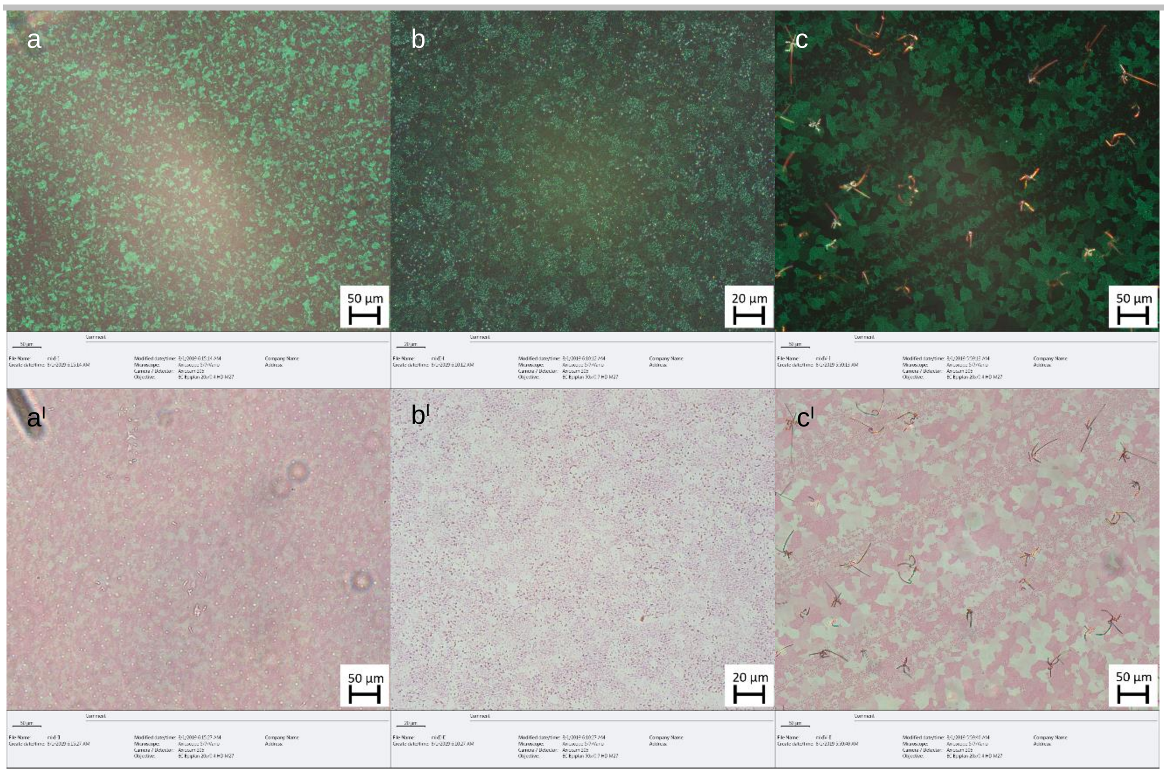


**Figure S7.** Transmission optical micrographs of mixed BODIPY films, *s*Bu- and IP-BODIPY (a, aI), *s*Bu- and *t*Bu-BODIPY (b, bI), IP-, *s*Bu- and *t*Bu-BODIPY (c, cI) with different directions of polarizers (top and bottom).

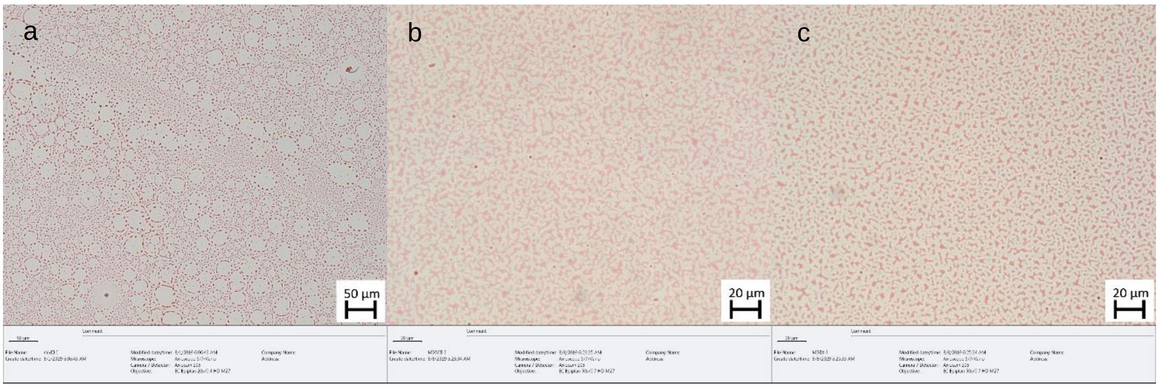


**Figure S8.** Transmission optical micrographs of mixed BODIPY films, IP- and *t*Bu-BODIPY (a), Et-, nBu- and IP-BODIPY (b), Et-, IP- and *t*Bu-BODIPY (c).

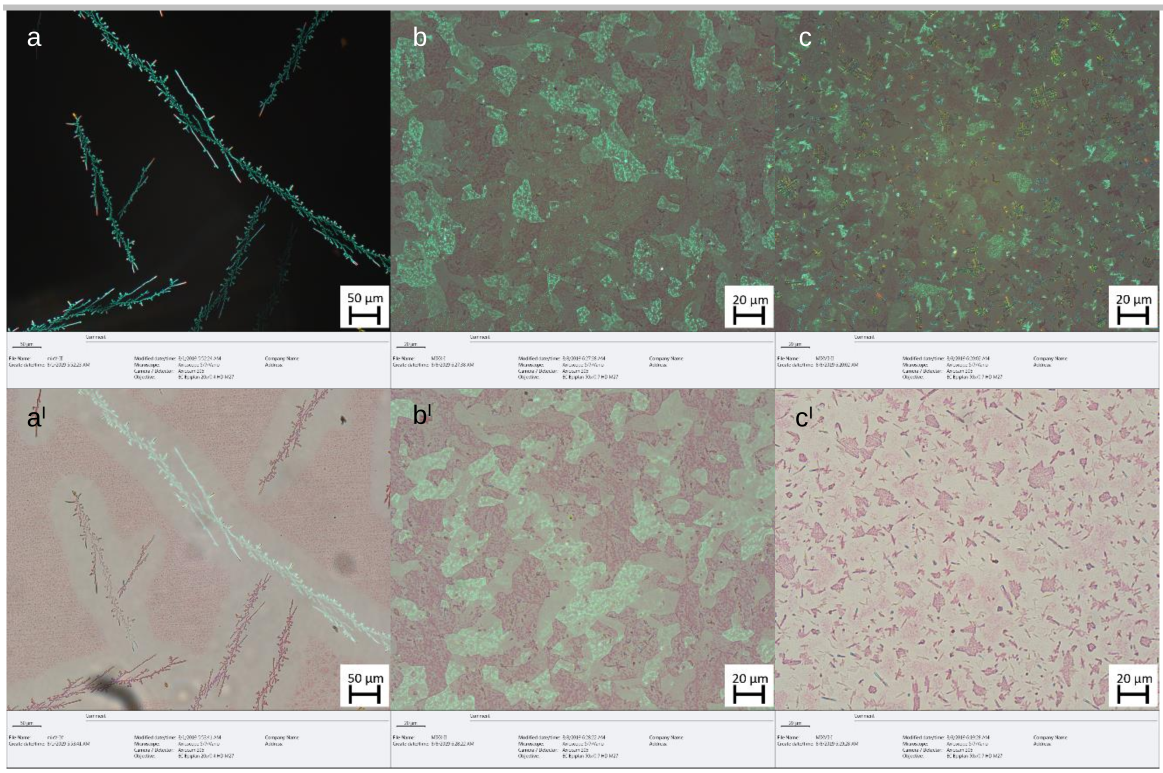


**Figure S9.** Transmission optical micrographs of mixed BODIPY films, Et-, nBu-, IP-, *s*Bu- and *t*Bu-BODIPY (a, $a^{I}$), nBu-, *s*Bu- and IP-BODIPY (b, $b^{I}$), nBu- and *s*Bu-BODIPY (c, $c^{I}$) with different directions of polarizers.

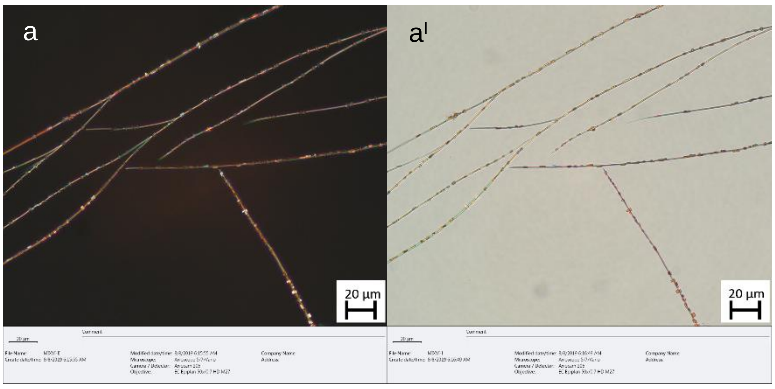


**Figure S10.** Transmission optical micrographs of mixed BODIPY film Et- and nBu-BODIPY with different directions of polarizers.

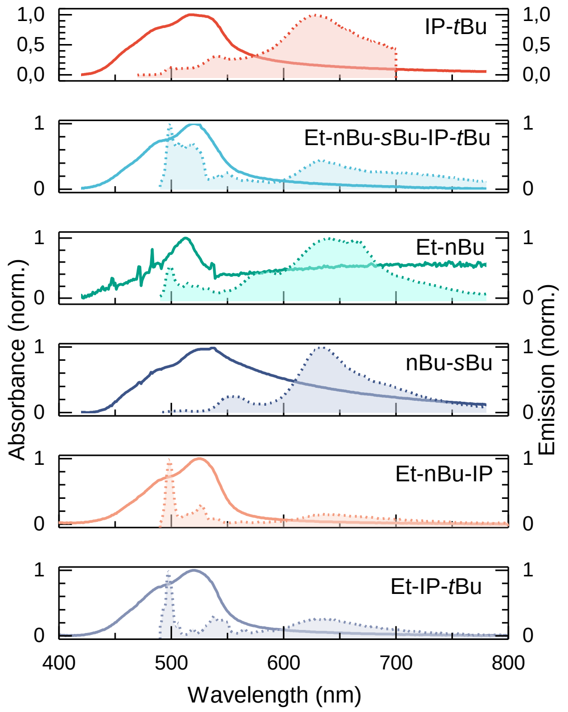


**Figure S11.** Absorbance and emission spectra of mixed films.

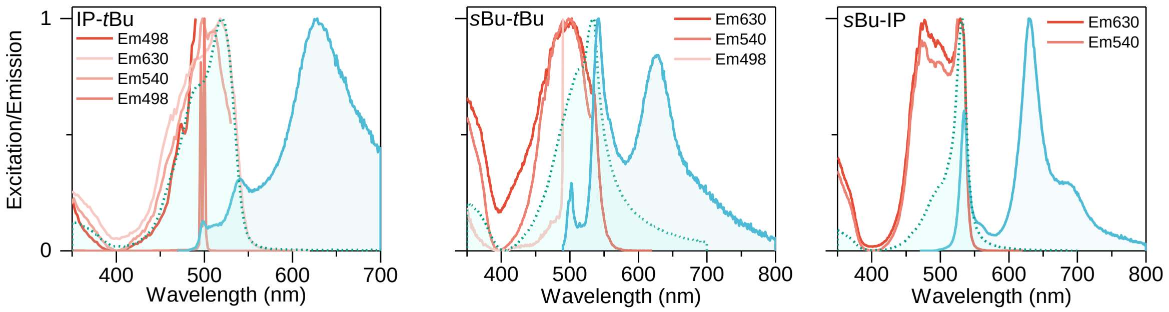


**Figure S12.** Absorbance (green), emission (blue) and excitation at different emission wavelengths (red) spectra of mixed films.

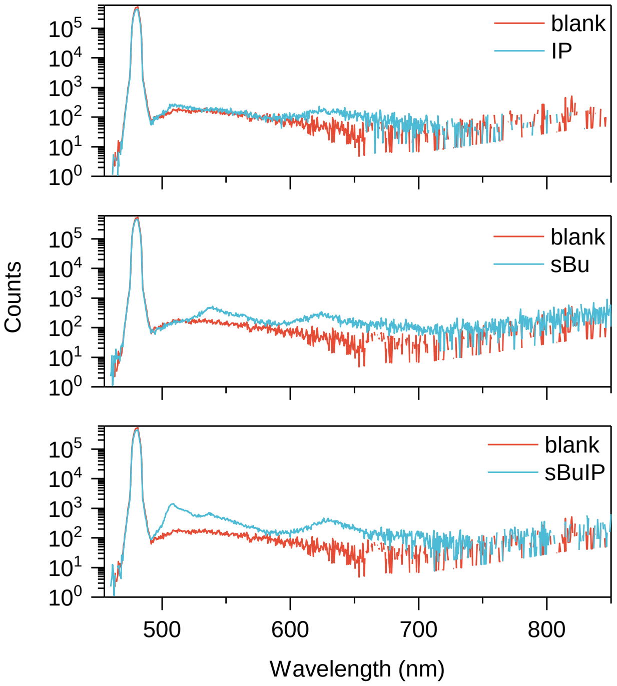


**Figure S13.** Emission quantum yields for the *s*Bu-IP blend as well as the individual components.

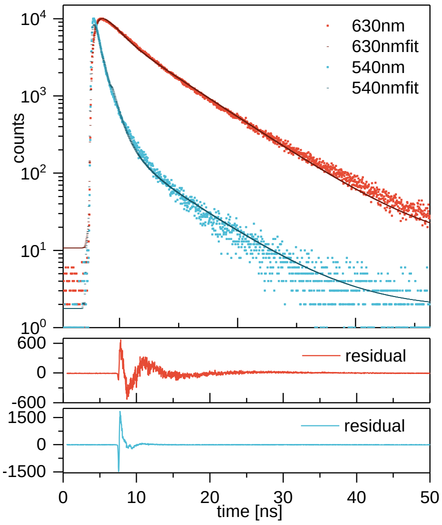


**Figure S14.** Emission decay of *s*Bu-*t*Bu at 540 nm and at 630 nm with the according fittings. The excitation wavelengths were 475 nm and 2048 channels were used to record the decay. Global fitting was done using three exponentials. The fitted lifetimes were $\tau_1$=0.416 ns, $\tau_2$=0.957 ns, and $\tau_3$=4.312 ns. The pre-factors to the lifetimes were -0.1885:0.1189:0.0602 and 0.0982:0.0157:0.0018 when recording at 630 nm and 540 nm, respectively.

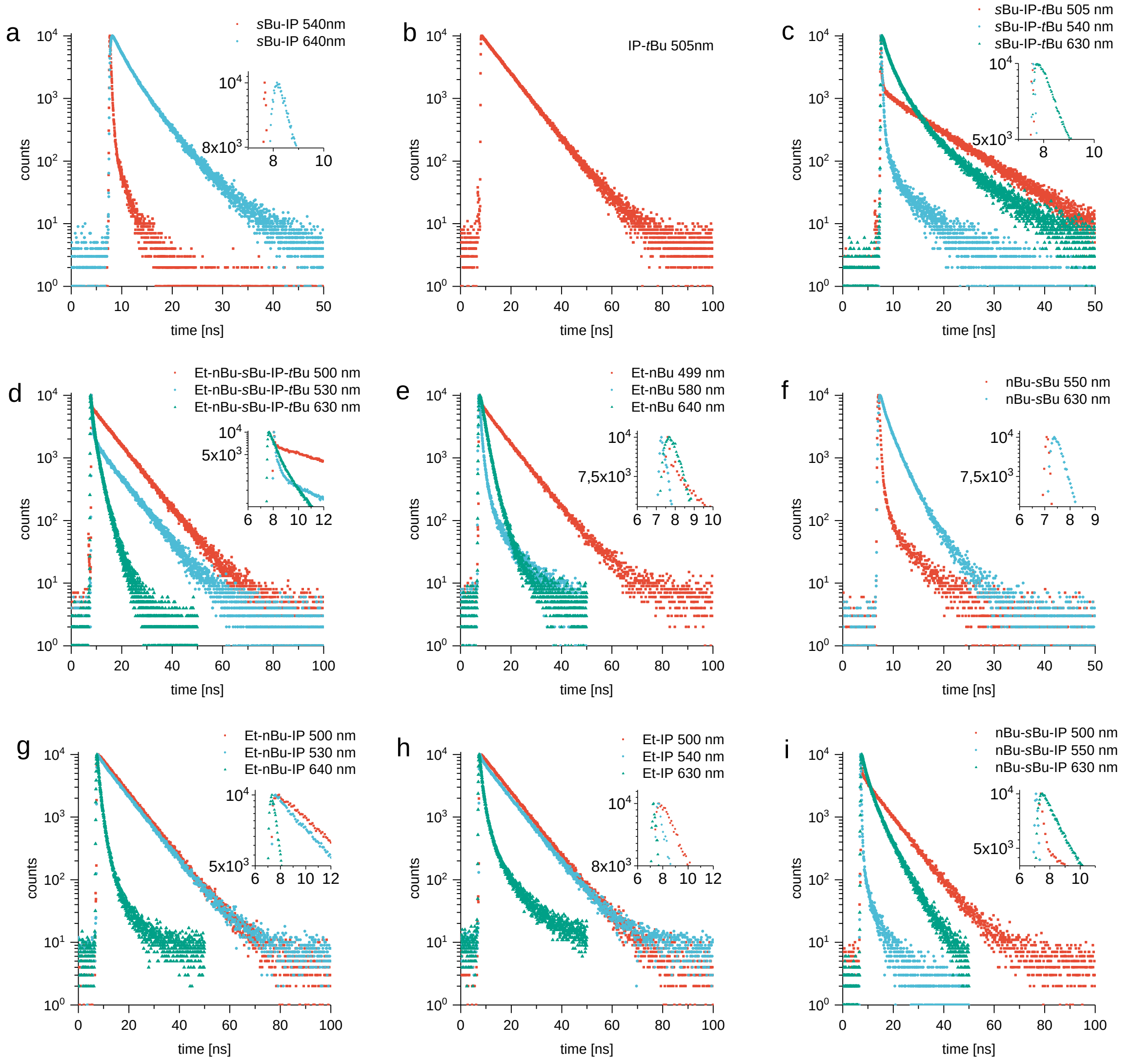


**Figure S15.** Emission decay of mixed films. The wavelength of excitation was 475 nm and 2048 channels were used when recording the decay.

## Synthesis

### 2-Acetylpyrrole

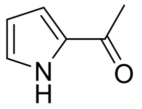

$POCl_3$ (2.8 mL, 30 mmol, 1.2 eq) was added drop-wise to *N,N*-dimethylacetamide (2.8 mL, 30 mmol, 1.2 eq) at 0 °C. The mixture was warmed up to room temperature and stirred until the Vielsmeier reagent was formed. The formed solid was dissolved in 1,2-dichloroethane (5 mL) and the solution was cooled down to 0 °C. Pyrrole (1.7 mL, 25 mmol, 1.0 eq) in 1,2-dichloroethane (10 mL) was added dropwise over a period of 20 min at 0 °C. After the addition was finished the reaction mixture was refluxed for 30 min and afterwards cooled down to room temperature. A solution of NaOAc (10.25 g, 125 mmol, 5.0 eq) in water was added to the reaction mixture and the mixture was refluxed again for 30 min. After the mixture was cooled to room temperature the 2 phase system was separated. The aqueous phase was extracted with DCM (3 x 25 mL). The combined organic phases were washed with water (1 x 50 mL), saturated $Na_2CO_3$ solution (2 x 50 mL) and brine (2 x 50 mL). The washed organic phase was dried over $Na_2SO_4$ and the solvent was removed under reduced pressure. The product was purified by column chromatography on $SiO_2$ using 20 % EtOAc in hexane as eluent which afforded the product as a white solid (2.29 g, 21 mmol, 84 %).
**$^1$H NMR** (400 MHz, $CDCl_3$) δ= 9.38 (br s, 1 H), 7.02 (ddd, *J* = 2.5, 1.3 Hz, 1 H), 6.91 (ddd, *J* = 3.8, 2.5, 1.3 Hz, 1 H), 6.28 (dt, *J* = 3.8, 2.5 Hz, 1 H), 2.43 (s, 3 H).
Recorded data are in accordance with the literature.[2]

### 2-Ethylpyrrole

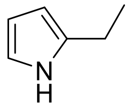

To a stirred suspension of $LiAlH_4$ (1.39 g, 36.7 mmol, 2 eq) in dry THF (18.4 mL(2 M solution in THF)) was added dropwise 2-acetylpyrrole (2.00 g, 18.3 mmol, 1 eq) in THF (25 mL) at 0 °C. The resulting solution was heated to reflux overnight. The reaction was quenched with saturated solution of $Na_2SO_4$. The insoluble solid was filtered off and washed with DCM. The organic phase was dried over $Na_2SO_4$ and the solvent was removed under reduced pressure. No further purification was needed. 2-Ethylpyrrole was afforded as a light yellow oily liquid. (1.40 g, 14.7 mmol, 80 %).
**$^1$H NMR** (400 MHz, $CDCl_3$) δ = 7.92 (s, 1H), 6.72 - 6.65 (m, 1H), 6.15 (q, *J* = 2.9 Hz, 1H), 5.96 - 5.93 (m, 1H), 2.66 (q, *J* = 7.6 Hz, 2H), 1.27 (t, *J* = 7.6 Hz, 3H).
Recorded data are in accordance with the literature.[3]

### *N,N*-Dimethylbutyramide

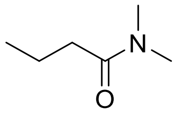

Dimethylamine (2 M solution in THF) (52,5 mL, 105 mmol, 1.5 eq) was diluted in DCM (0.2 M) and $NEt_3$ (29.4 mL, 210 mmol, 3 eq) was added to the solution. Butyryl chloride (7.35 mL, 70 mmol, 1 eq) in DCM was added dropwise at 0 °C. The reaction mixture was quenched with a saturated aqueous $NH_4Cl$ solution after stirring at room temperature for 18 h. The phases were separated and the aqueous phase was extracted with DCM (2 x 50 mL). The combined organic phases were dried over $Na_2SO_4$ and the solvent was removed under reduced pressure. The product was purified using column chromatography ($SiO_2$, 0 - 20 % DMA mix/DCM). The product was obtained as a white solid (6.55 g, 56.91 mmol, 81 %).
DMA mix: 90% DCM, 10% MeOH and 0.2% $Et_3N$.
**$^1$H NMR** (400 MHz, $CDCl_3$) δ = 2.97 (s, 3H), 2.90 (s, 3H), 2.25 (t, *J* = 7.4 Hz, 1H), 1.62 (h, *J* = 7.4 Hz, 1H), 0.92 (t, *J* = 7.4 Hz, 3H).
Recorded data are in accordance with the literature.[4]

**1-(1H-Pyrrol-2-yl)butan-1-one**

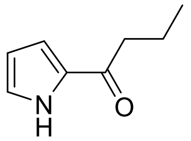

$POCl_3$ (2.81 mL, 30 mmol, 1.2 eq.) was added slowly to *N,N* -dimethylbutyramide (3.45 g, 30 mmol, 1.2 eq.) at 0 °C. The reaction mixture was stirred for 1 h at room temperature. Afterwards the reaction mixture was cooled down to 0 °C and pyrrole (1.81 mL, 25 mmol, 1 eq) dissolved in 1,2-dichloroethane (5 mL) was added to dropwise over a period of 15 min. The resulting mixture was allowed to warm up to room temperature and stirred for 18 h. An aqueous solution of NaOAc (10.25 g, 125 mmol, 5 eq) was added to the reaction mixture and the 2 phase system was left to stir for 1 h at room temperature. The reaction mixture was extracted with DCM (3 x 2 mL) and the combined organic phases were washed with $H_2O$ (2 x 50 mL), $Na_2CO_3$ (2 x 50 mL) and brine (2 x 50 mL). The washed organic phases were subsequently dried over $Na_2SO_4$ and the solvent was removed under reduced pressure. Flash chromatography on $SiO_2$ using 5 – 20% EtOAc in hexane as an eluent afforded the product as colourless crystals (2.87 g, 20.9 mmol, 84 %).
**$^1$H NMR** (400 MHz, $CDCl_3$ ) δ = 9.66 (s, 1H), 7.02 (td, J = 2.5, 1.3 Hz, 1H), 6.91 (ddd, J = 3.8, 2.5, 1.3 Hz, 1H), 6.27 (dt, J = 3.8, 2.5 Hz, 1H), 2.74 (t, J = 7.4 Hz, 2H), 1.75 (h, J = 7.4 Hz, 2H), 0.99 (t, J = 7.4 Hz, 3H).
**$^{13}$C NMR** (101 MHz, $CDCl_3$ ) δ = 191.2, 132.1, 124.7, 116.3, 110.4, 39.9, 18.7, 14.0.
Recorded data are in accordance with the literature.[5]

**2-Butylpyrrole**

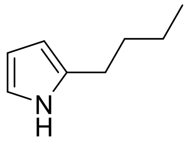

1-(1H-pyrrol-2-yl)butan-1-one (20 mmol, 2.87 g, 1 eq) was dissolved in isopropanol (150 mL) and stirred for 5 min. $NaBH_4$ (80 mmol, 3.03 g, 4 eq) was added and the reaction mixture was refluxed for 18 h. Afterwards the reaction mixture was concentrated under reduced pressure. The concentrated mixture was subsequently taken up in $H_2O$ and $Et_2O$ the aqueous phase was extracted with $Et_2O$ (3 x 50 mL). The combined organic phases were washed with $H_2O$, dried over $Na_2SO_4$ and the solvent was removed under reduced pressure. The product was used without further purification (2.15 g, 17.4 mmol, 87 %)
**$^1$H NMR** (400 MHz, $CDCl_3$ ) δ = 7.89 (s, 1H), 6.67 (td, *J* = 2.6, 1.6 Hz, 1H), 6.14 (q, *J* = 2.8 Hz, 1H), 5.91 - 5.94 (m, 1H), 2.61 (t, *J* = 7.4 Hz, 2H), 1.62 (ddt, *J* = 8.8, 7.4, 6.3 Hz, 2H), 1.39 (dq, *J* = 14.5, 7.4 Hz, 2H), 0.94 (t, *J* = 7.4 Hz, 3H).
Recorded data are in accordance with the literature. [6]

**General Procedure A: to make the branched alkylated pyrroles a modified literature procedure was used** [7]

Magnesium powder (1 eq) was stirred in dry $Et_2O$ at r.t. MeI (1 eq) in dry $Et_2O$ was added dropwise to the reaction mixture over 30 min. The mixture started to reflux and was left to reflux for 1.5 h after the addition was finished. The reaction mixture was cooled down to room temperature and pyrrole (1 eq) in dry $Et_2O$ was added over 30 min. The mixture was left to reflux for 1 h after the addition of pyrrole was finished. The alkylbromide (1 eq) was added dropwise to the reaction of the formed pyrrole-grignard reagent. The resulting solution was refluxed for 24 h and then cooled down to room temperature. The cooled reaction mixture was quenched using a saturated $NH_4Cl$ solution. The 2 phases were separated and the aqueous phase was extracted using $Et_2O$. The combined organic phases were dried over $Na_2SO_4$ and the solvent was removed under reduced pressure. The crude mixture was purified via vacuum distillation. The purification afforded a mixture of 2-alkyl and 3-alkyl substituted pyrrole. The mixture was used for the next step without further purification.

**2-*sec*Butylpyrrole**

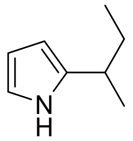

The compound was synthesized following the general **Procedure A** using pyrrole (7.2 mL, 100 mmol) and 1-bromo-1-methylpropane (10.9 mL, 100 mmol). The crude mixture was purified via vacuum distillation affording a mixture of 2- and 3-*sec*butylpyrrole (4.20 g, 34.1 mmol, 34 %). The mixture of 2- and 3-*sec*butylpyrrole contained 66 % 2-*sec*butylpyrrole.

**2-Isopropylpyrrole**

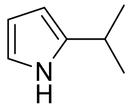


The compound was synthesized following the general **Procedure A** using pyrrole (7.2 mL, 100 mmol) and 2-bromopropane (9.6 mL, 100 mmol). The crude mixture was purified via vacuum distillation affording a mixture of 2- and 3-isopropylpyrrole (1.14 g, 10.5 mmol, 11 %).The mixture of 2-isopropyl and 3-isopropyl substituted pyrrole contains 70 % 2-isopropylpyrrole.

**2-*tert*Butylpyrrole**

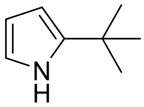


The compound was synthesized following the general **Procedure A** using pyrrole (7.2 mL, 100 mmol) and 2-bromo-2-methylpropane (11.5 mL, 100 mmol). The crude mixture was purified via vacuum distillation affording a mixture of 2- and 3-*tert*butylpyrrole (4.00 g, 32.5 mmol, 33 %). The mixture of 2- and 3-*tert*butylpyrrole contained 50 % 2-*tert*butylpyrrole.

**2-Butyl-5-formylpyrrole**

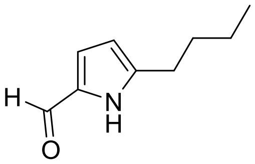


$POCl_3$ (0.21 mL, 2.2 mmol, 1.1 eq) in dry toluene (0.3 mL) was added dropwise to *N,N*-dimethylformamide (0.17 mL, 2.2 mmol, 1.1 eq) in dry toluene (0.4 mL) at 0 °C. The mixture was warmed up to room temperature and stirred until the Vielsmeier reagent was formed. 2-Butylpyrrole (250 mg, 2.0 mmol, 1.0 eq) in DMF (0.5 mL) was added drop-wise over a period of 20 min at 0 °C. After the addition was finished, the reaction mixture was stirred for 1 h at room temperature. The reaction mixture was poured in iced water (20 mL) and $NaOH_{aq}$ was added until the solution was alkaline. The mixture was extracted with $CHCl_3$ (3 x 20 mL) and afterwards dried with $Na_2SO_4$. The solvent was removed under reduced pressure and the crude product was purified by column chromatography on silica using 20 % EtOAc in hexane as an eluent which afforded the product as a light yellow solid (254 mg, 1.68 mmol, 84 %).
**$^1$H NMR** (400 MHz, $CDCl_3$) δ = 9.36 (s, 1H), 6.88 (dd, *J* = 3.8, 2.4 Hz, 1H), 6.07 (ddt, *J* = 3.8, 2.5, 0.6 Hz, 1H), 2.65 (t, *J* = 7.5 Hz, 2H), 1.67 - 1.59 (m, 3H), 1.36 (dq, *J* = 14.6, 7.4 Hz, 2H), 0.92 (t, *J* = 7.3 Hz, 3H).
Recorded data are in accordance with the literature. [8]

**General Procedure B: for the Vielsmeier-Haack Formylation a literature procedure was used [9]**

$POCl_3$ (1.2 eq) was added dropwise to DMF (1.2 eq) at 0 °C. The mixture was left to warm up to room temperature and stirred until the Vielsmeier reagent was formed. The formed solid was subsequently dissolved in 1,2-dichloroethane and the solution was cooled down to 0 °C. The alkylpyrrole (1.0 eq) in 1,2-dichloroethane was afterwards added dropwise over a period of 30 min at 0 °C. After the addition was finished, the reaction mixture was refluxed for 30 min and cooled to room temperature. An aqueous solution of NaOAc (5.0 eq) was added and the mixture was again refluxed for 30 min. After cooling down to room temperature the 2 phase system was separated and the aqueous phase was extracted with DCM. The combined organic phases were washed with $H_2O$, saturated $Na_2CO_3$ solution and brine afterwards dried with $Na_2SO_4$ and the solvent was removed under reduced pressure. The product was purified by column chromatography on silica gel using 0 - 20 % EtOAc in hexane as an eluent.

**2-Ethyl-5-formylpyrrole**

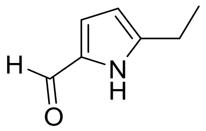

The compound was synthesized following the general **Procedure B** using 2-Ethylpyrrole (1.25 g, 13 mmol). The product was purified by column chromatography on silica gel using 20 % EtOAc in hexane as an eluent. The product was afforded as an off-white solid (1.14 g, 9.25 mmol, 71%).
$^{1}$**H NMR** (400 MHz, $CDCl_3$ ) δ = 10.14 (br s, 1H), 9.36 (s, 1H), 6.91 (dd, *J* = 3.8, 2.5 Hz, 1H), 6.09 (dd, *J* = 3.8, 2.5 Hz, 1H), 2.72 (q, *J* = 7.6 Hz, 2H), 1.28 (t, *J* = 7.6 Hz, 3H).
Obtained data are in agreement with the literature.[9]

**2-Isopropyl-5-formylpyrrole**

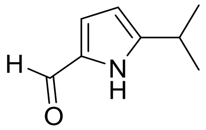

The compound was synthesized following the general **Procedure B** using a mixture of 3- and 2-isopropylpyrrole (containing 70 % 2-isopropylpyrrole) (218 mg, 2.00 mmol). The product was purified by column chromatography on silica gel using 0 - 20 % EtOAc in hexane as an eluent. The product was afforded as an off-white oily liquid (135 mg, 0.99 mmol, 74%).
$^{1}$**H NMR** (400 MHz, $CDCl_3$) δ = 9.62 (s, 1H, NH), 9.38 (s, 1H, CHO), 6.90 (ddd, *J* = 3.9, 2.5, 0.4 Hz, 1H, pyrrole), 6.09 (ddd, *J* = 3.8, 2.5, 0.6 Hz, 1H, pyrrole), 3.01 (hept, *J* = 6.9 Hz, 1H, CH), 1.30 (s, 3H, $CH_3$), 1.29 (s, 3H, $CH_3$).
$^{13}$**C NMR** (101 MHz, $CDCl_3$) δ = 178.3, 149.9, 131.8, 123.2, 107.4, 27.6, 22.2.
**HRMS** (ESI+) m/z calcd. for $(M+H)^+$ $C_8H_{11}NO$: 138.186; found: 138.103.

**2-*sec*Butyl -5-formylpyrrole**

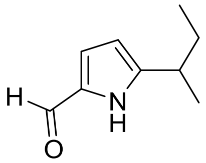

The compound was synthesized following the general **Procedure B** using a mixture of 3- and 2-*sec*butylpyrrole (1.72 g, 14.0 mmol). The product was purified by column chromatography on silica gel using 0 - 20 % EtOAc in hexane as an eluent. The product was afforded as an off-white oily liquid (1.08 g, 7.14 mmol, 73%).
$^{1}$**H NMR** (400 MHz, $CDCl_3$) δ = 10.14 (s, 1H, NH), 9.38 (s, 1H, CHO), 6.94 (dd, *J* = 3.9, 2.4 Hz, 1H, pyrrole), 6.08 (ddd, J = 3.9, 2.4, 0.6 Hz, 1H, pyrrole), 2.86 (h, J = 7.0 Hz, 1H, CH), 1.76 - 1.55 (m, 2H, $CH_2$), 1.28 (d, J = 7.0 Hz, 3H, $CH_3$), 0.85 (t, J = 7.5 Hz, 3H, CH).
$^{13}$**C NMR** (101 MHz, $CDCl_3$) δ = 178.2, 149.5, 131.8, 123.6, 108.0, 34.7, 29.9, 19.9, 11.7
**HRMS** (ESI+) m/z calcd. for $(M+H)^+$ $C_9H_{13}NO$: 152.212; found: 152.107.

**2-*tert*Butyl-5-formylpyrrole**

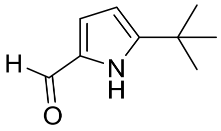

The compound was synthesized following the general **Procedure B** using a mixture of 3- and 2-*tert*butylpyrrole (containing 50 % of 2-*tert*butylpyrrole) (246 mg, 2.0 mmol). The product was purified by column chromatography on silica gel using 0 - 15 % EtOAc in hexane as an eluent. The product was afforded as an off-white oily liquid (107 mg, 0.71 mmol, 71 %).
$^{1}$**H NMR** (400 MHz, $CDCl_3$) δ = 10.15 (s, 1H), 9.38 (s, 1H), 6.87 (dd, J = 3.9, 2.5 Hz, 1H), 6.09 (dd, J = 3.9, 2.5 Hz, 1H), 1.33 (s, 9H).
$^{13}$**C NMR** (101 MHz, $CDCl_3$) δ = 171.4, 136.0, 130.0, 128.0, 118.1, 35.0, 30.5, 30.5, 30.4.
Obtained NMR data are in agreement with the literature.[10]

**α-EthylBODIPY**

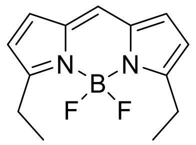


2-Ethyl-5-formylpyrrole (246 mg, 2 mmol, 1 eq) was dissolved in DCM/pentane [2/1] (1 mL) and the solution was cooled down to 0 °C. $POCl_3$ (0.18 mL, 2 mmol, 1 eq) was added dropwise over a period of 3 min. The resulting mixture was stirred at room temperature for 4 h. $Et_3N$ (2.78 mL, 20 mmol, 10 eq) was added dropwise over 5 min at room temperature and the reaction mixture was left to stir for 15 min before cooling the solution down to 0 °C. $BF_3{\cdot}OEt_2$ (2.73 mL, 22 mmol, 11 eq) was added dropwise at 0 °C and the reaction mixture was afterwards stirred at room temperature for 1.5 h. The reaction mixture was poured in $Et_2O$ (100 mL) and the organic phase was washed with $H_2O$ (4 x 50 mL). The organic phase was dried over $Na_2SO_4$ and the solvent was removed under reduced pressure. The product was purified via column chromatography on $SiO_2$ using 10-25 % DCM in hexane as eluent. The pure product was obtained as a red crystalline solid (28 mg, 0.11 mmol, 11%)
**$^1$H NMR** (400 MHz, $CDCl_3$) δ = 7.07 (s, 1H), 6.96 (d, *J* = 4.2 Hz, 2H), 6.34 (d, *J* = 4.2 Hz, 2H), 3.02 (q, *J* = 7.6 Hz, 4H), 1.32 (t, *J* = 7.6 Hz, 6H).
**$^{13}$C NMR** (101 MHz, $CDCl_3$) δ = 164.1, 134.4, 130.1, 127.1, 117.4, 22.1, 12.7.
Obtained data are in agreement with the literature.[9]

**α-*tert*ButylBODIPY**

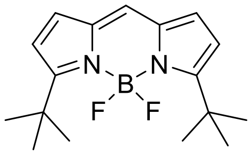


2-*tert*Butyl-5-formylpyrrole (302 mg, 2.0 mmol, 1 eq) was dissolved in DCM (10 mL) and the solution was cooled down to 0 °C. $POCl_3$ (0.23 mL, 2.4 mmol, 1.2 eq) was added dropwise over a period of 5 min. The resulting mixture was stirred at room temperature for 6 h. $Et_3N$ (1.40 mL, 10 mmol, 5 eq) was added dropwise over 5 min at 0 °C and the reaction mixture was left to stir for 20 min before $BF_3{\cdot}OEt_2$ (2.00 mL, 16.0 mmol, 8 eq) was added dropwise at 0 °C. The reaction mixture was afterwards slowly warmed to room temperature and stirred for 12 h. The reaction mixture was passed through a short pad of silica and eluted with DCM. The solvent was evaporated and the residue was again dissolved in DCM (25 mL). $H_2O$ was added to the organic phase and the two-phase mixture was stirred at room temperature for 2 h. Afterwards the organic phase was washed with $H_2O$ (3 x 50 mL). The organic phase was dried over $Na_2SO_4$ and the solvent was removed under reduced pressure. After purification, using column chromatography ($SiO_2$, 5 – 20 % DCM in hexane), the product was obtained as a red crystalline solid (42 mg, 0.15 mmol, 15 %).
**$^1$H NMR** (400 MHz, $CDCl_3$) δ = 7.10 (s, 1H), 6.97 (d, *J* = 4.3 Hz, 2H), 6.49 (dd, *J* = 4.3, 0.8 Hz, 2H), 1.52 (s, 18H).
**$^{13}$C NMR** (101 MHz, $CDCl_3$) δ = 71.4, 136.0, 129.9, 127.9, 118.1, 35.0, 30.5, 30.5, 30.4.
Obtained data are in agreement with the literature.[11]

**General Procedure C: for the BODIPY condensation**

2-Alkyl-5-formylpyrrole (1 eq) was dissolved in DCM/pentane [2/1] and the solution was cooled down to 0 °C. $POCl_3$ (2 eq) was added dropwise over a period of 3 min. The resulting mixture was stirred at room temperature for 2.5 h. $Et_3N$ (6 eq) was added dropwise over 5 min at room temperature and the reaction mixture was left to stir for 15 min before cooling the solution down to 0 °C. $BF_3{\cdot}OEt_2$ (9 eq) was added dropwise at 0 °C and the reaction mixture was afterwards stirred at room temperature for 30 min. Repeatedly, $Et_3N$ (6 eq) was added dropwise over 5 min at room temperature and the reaction mixture was left to stir for 15 min before cooling the solution down to 0 °C. $BF_3{\cdot}OEt_2$ (9 eq) was added dropwise at 0 °C and the reaction mixture was afterwards stirred at room temperature for 1 h. The reaction mixture was poured in $Et_2O$ and the organic phase was washed with $H_2O$. The organic phase was dried over $Na_2SO_4$ and the solvent was removed under reduced pressure. The product was purified via column chromatography on $SiO_2$ using 10-20 % DCM in hexane as eluent.

**α-ButylBODIPY**

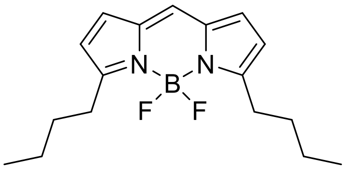


The compound was synthesized following the general **Procedure C** using 2-butyl-5-formylpyrrole (0.65 g, 4.3 mmol, 1 eq). The product was purified via column chromatography on $SiO_2$ using 10-15 % DCM in hexane as eluent. The product was obtained as a red crystalline solid (63.8 mg, 0.21 mmol, 10 %).

**M.p**: 89 - 91 °C

**$^1$H NMR** (400 MHz, $CDCl_3$) δ = 7.05 (s, 1H), 6.94 (d, *J* = 3.9 Hz, 2H), 6.32 (d, *J* = 3.9 Hz, 2H), 2.99 (t, *J* = 7.6 Hz, 5H), 1.71 (dt, *J* = 15.3, 7.6 Hz, 5H), 1.45 (dd, *J* = 15.3, 7.6 Hz, 5H), 0.95 (t, *J* = 7.6 Hz, 7H).

**$^{13}$C NMR** (101 MHz, $CDCl_3$) δ = 163.1, 134.3, 129.9, 126.8, 117.9, 30.6, 28.5, 22.7, 13.9.

**HRMS** (ESI+) m/z calcd. for $(M+H)^+$ $C_{17}H_{23}BF_2N_2$: 305.198; found: 305.201.

**IR** (in $cm^{-1}$): 2954, 2926, 2854, 1609, 1486, 1439, 1415, 1324, 1256, 1123, 1083, 1058, 1024, 980.

**α-*sec*ButylBODIPY**

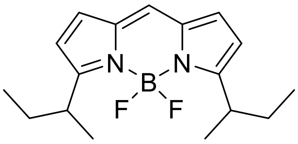


The compound was synthesized following the general **Procedure C** using 2-*sec*butyl-5-formylpyrrole (85 mg, 0.56 mmol, 1 eq). The product was purified via column chromatography on $SiO_2$ using 10-15 % DCM in hexane as eluent. The product was obtained as an orange crystalline solid (39 mg, 0.13 mmol, 49 %).

**M.p**: 207 - 208 °C

**$^1$H NMR** (400 MHz, $CDCl_3$) δ = 7.07 (s, 1H, CH-bridge), 6.98 (d, *J* = 4.0 Hz, 2H, pyrrole), 6.35 (d, *J* = 4.5 Hz, 2H, pyrrole), 3.37 (h, *J* = 7.2 Hz, 2H, CH), 1.78 - 1.58 (m, 4H, $CH_2$), 1.29 (dd, *J* = 6.9, 0.8 Hz, 6H, $CH_3$), 0.93 (td, *J* = 7.4, 2.0 Hz, 6H, $CH_3$).

**$^{13}$C NMR** (101 MHz, $CDCl_3$) δ = 168.2, 133.6, 130.0, 127.0, 115.5, 35.1, 29.8, 20.7, 12.0.

**HRMS** (ESI+) m/z calcd. for $(M+H)^+$ $C_{17}H_{23}BF_2N_2$: 305.198; found: 305.196.

**IR** (in $cm^{-1}$): 2964, 2930, 2870, 1613, 1492, 1432, 1334, 1267, 1236, 1159, 1106, 1049, 974.

**α-IsopropylBODIPY**

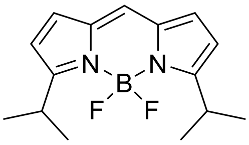


The compound was synthesized following the general **Procedure C** using 2-*sec*butyl-5-formylpyrrole (135 mg, 1.00 mmol, 1 eq). The product was purified via column chromatography on $SiO_2$ using 10-15% DCM in hexane as eluent. The product was obtained as a red crystalline solid (39 mg, 0.14 mmol, 28 %).

**M.p**: 115 - 117 °C

**$^1$H NMR** (400 MHz, $CDCl_3$) δ = 7.09 (s, 1H, CH-bridge), 6.98 (d, *J* = 4.2 Hz, 2H, pyrrole), 6.39 (dd, *J* = 4.2, 0.5 Hz, 2H, pyrrole), 3.59 (hept, *J* = 6.8 Hz, 2H, CH), 1.33 (s, 6H, $CH_3$), 1.31 (s, 6H, $CH_3$).

**$^{13}$C NMR** (101 MHz, $CDCl_3$) δ = 168.9, 133.7, 130.1, 127.4, 115.3, 28.1, 22.7.

**HRMS** (ESI+) m/z calcd. for $(M+H)^+$ $C_{15}H_{19}BF_2N_2$: 277.168; found: 277.168.

**IR** (in $cm^{-1}$): 2968, 2932, 2875, 1609, 1493, 1435, 1331, 1252, 1159, 1106, 1041, 977.

## NMR spectra

### 2-Acetylpyrrole

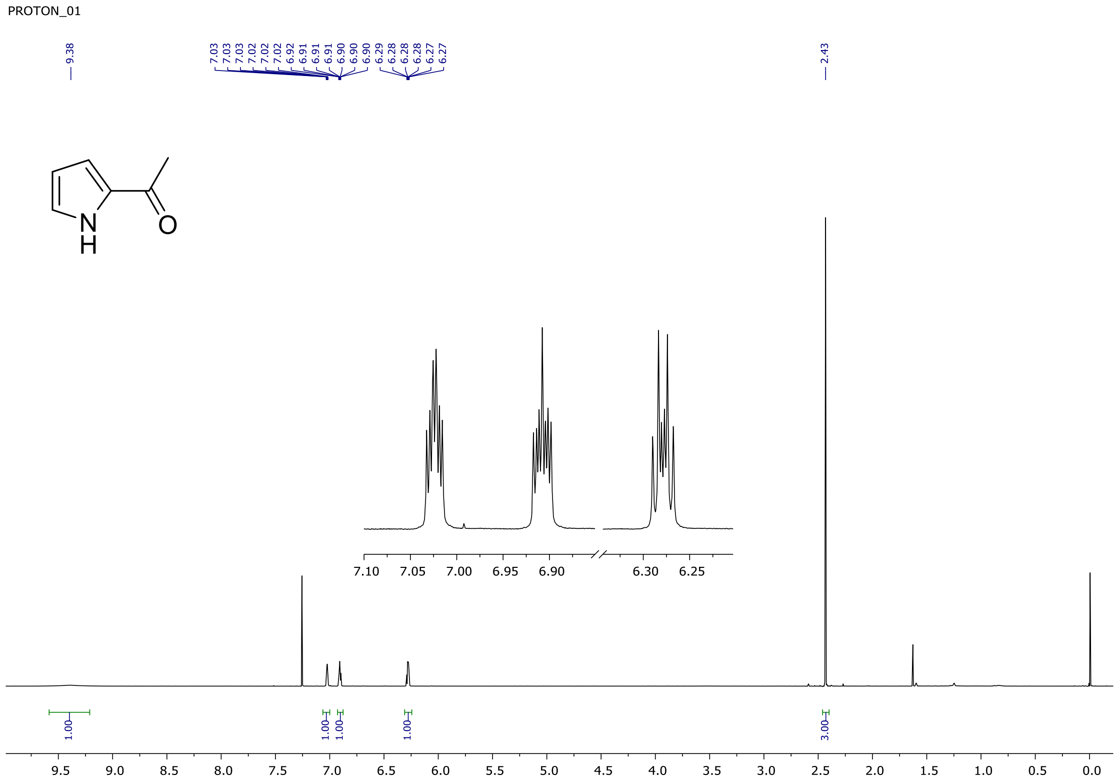


**Figure S16.** $^{1}$H-NMR of 2-Acetylpyrrole.

### 2-Ethylpyrrole

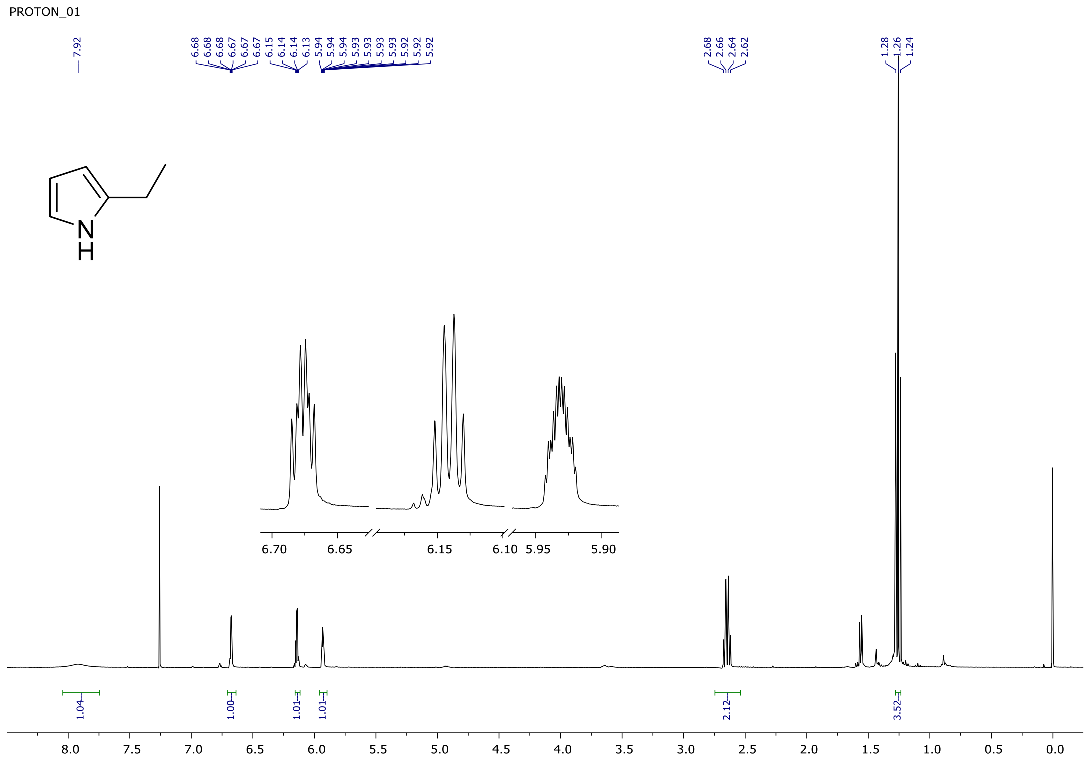


**Figure S17.** $^{1}$H-NMR of 2-Ethylpyrrole.

### *N,N*-Dimethylbutyramide

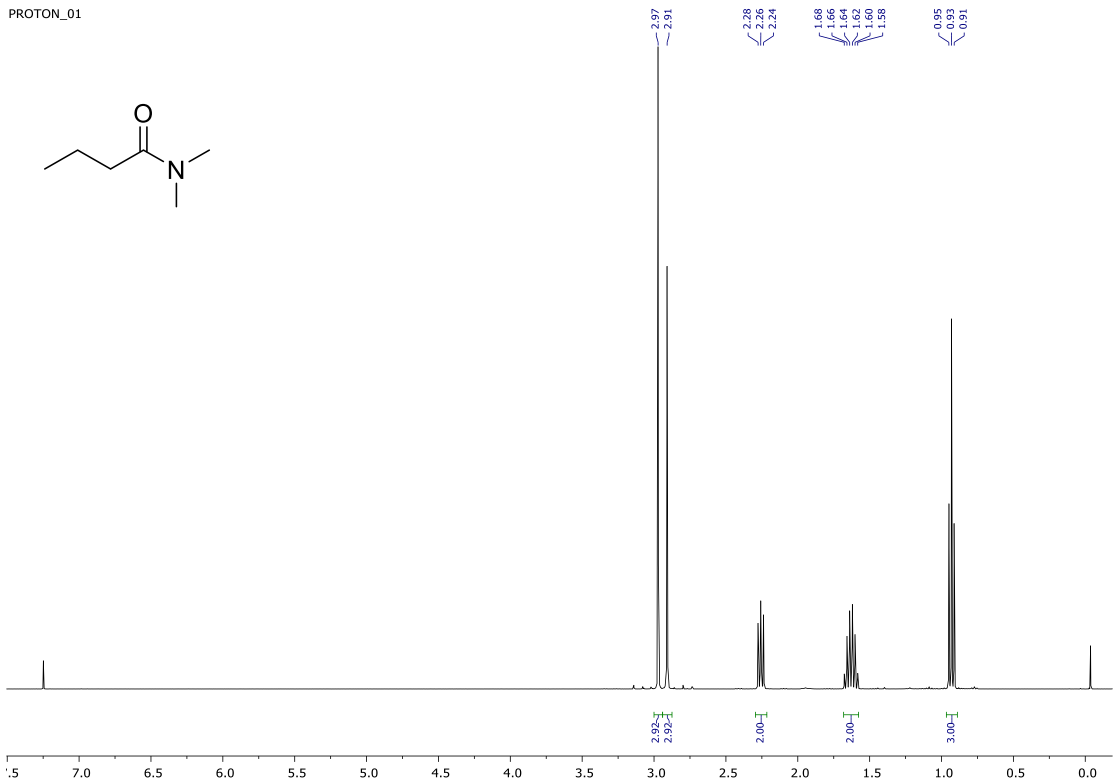


**Figure S18.** $^1$H-NMR of *N*,*N*-Dimethylbutyramide.

### 1-(1H-Pyrrol-2-yl)butan-1-one

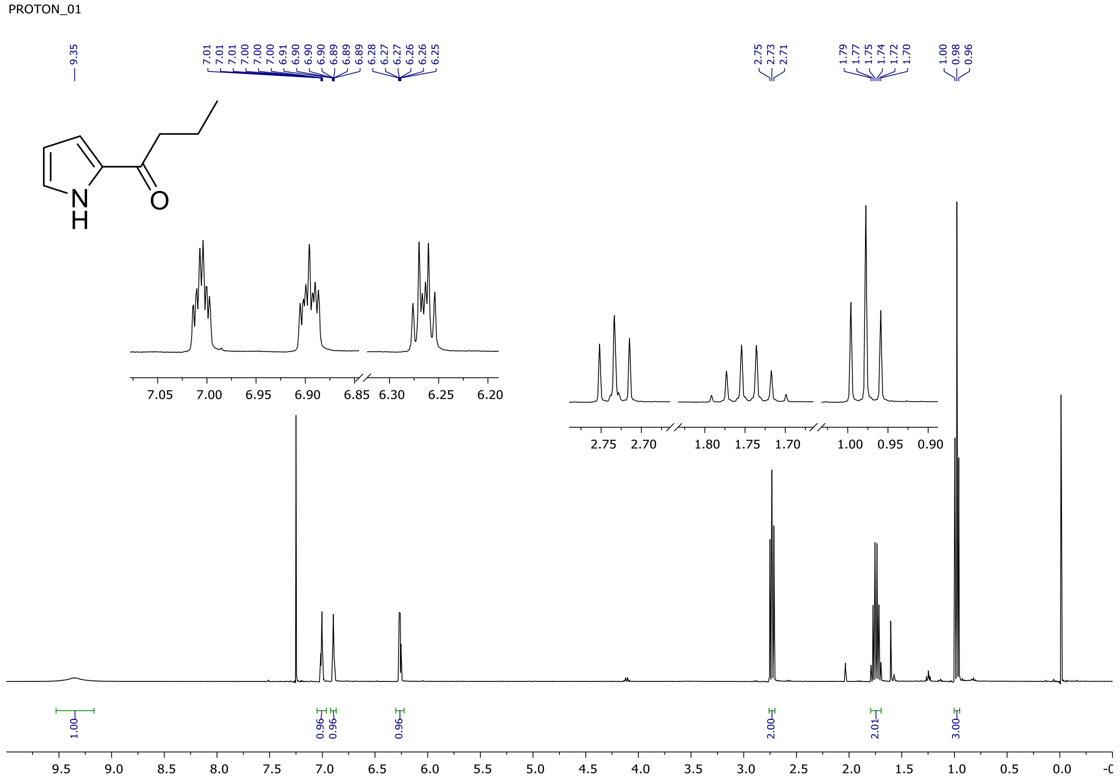


**Figure S19.** $^1$H-NMR of 1-(1H-Pyrrol-2-yl)butan-1-one.

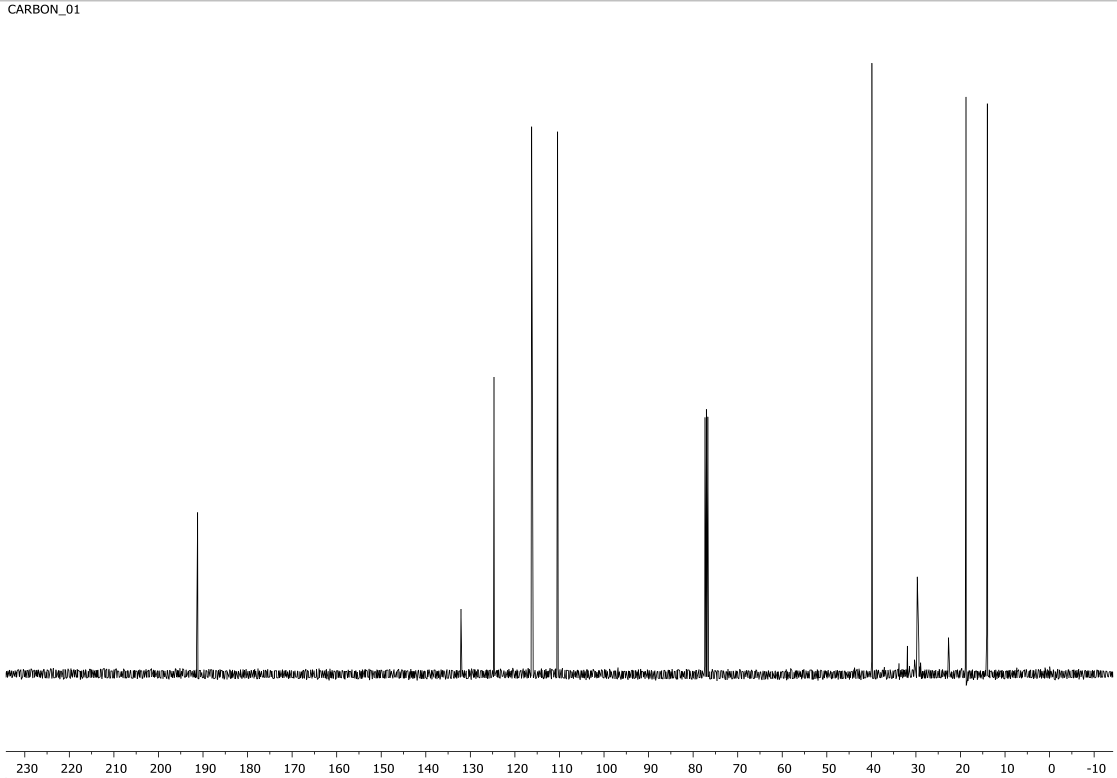


**Figure S20.** $^{13}$C-NMR of 1-(1H-Pyrrol-2-yl)butan-1-one.

## 2-Butylpyrrole

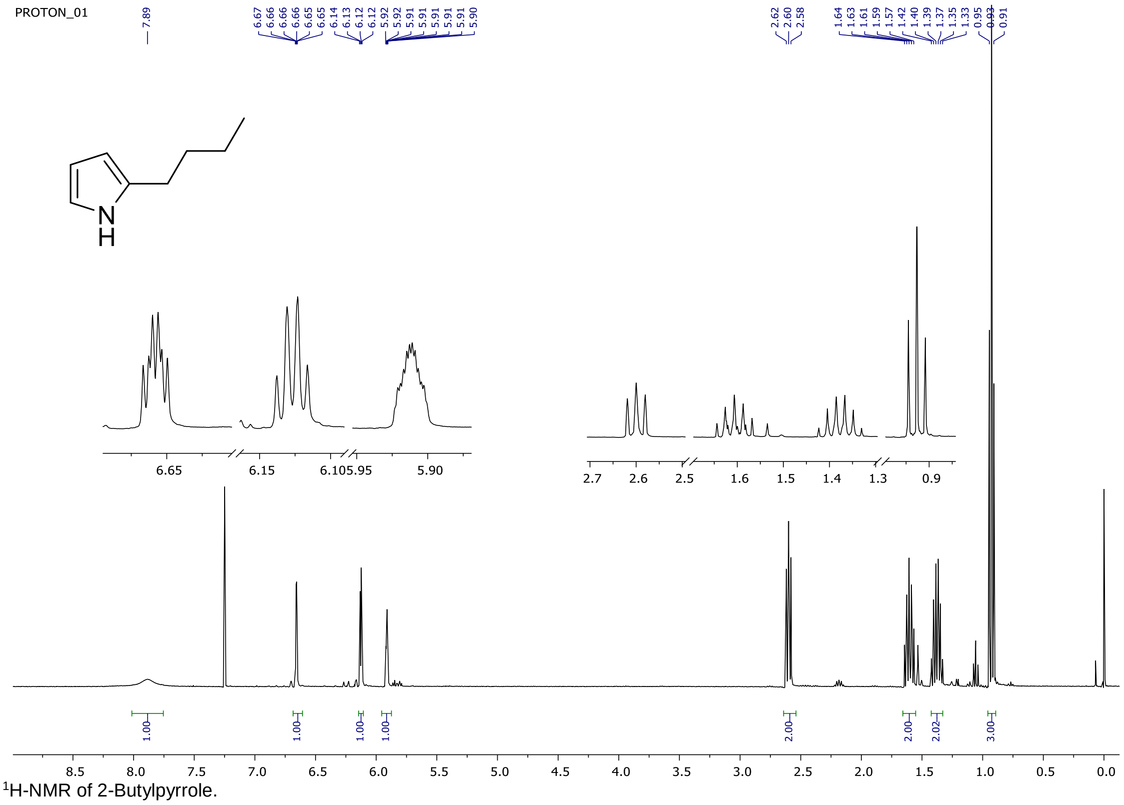


**Figure S21.** $^{1}$H-NMR of 2-Butylpyrrole.

**2-Butyl-5-formylpyrrole**

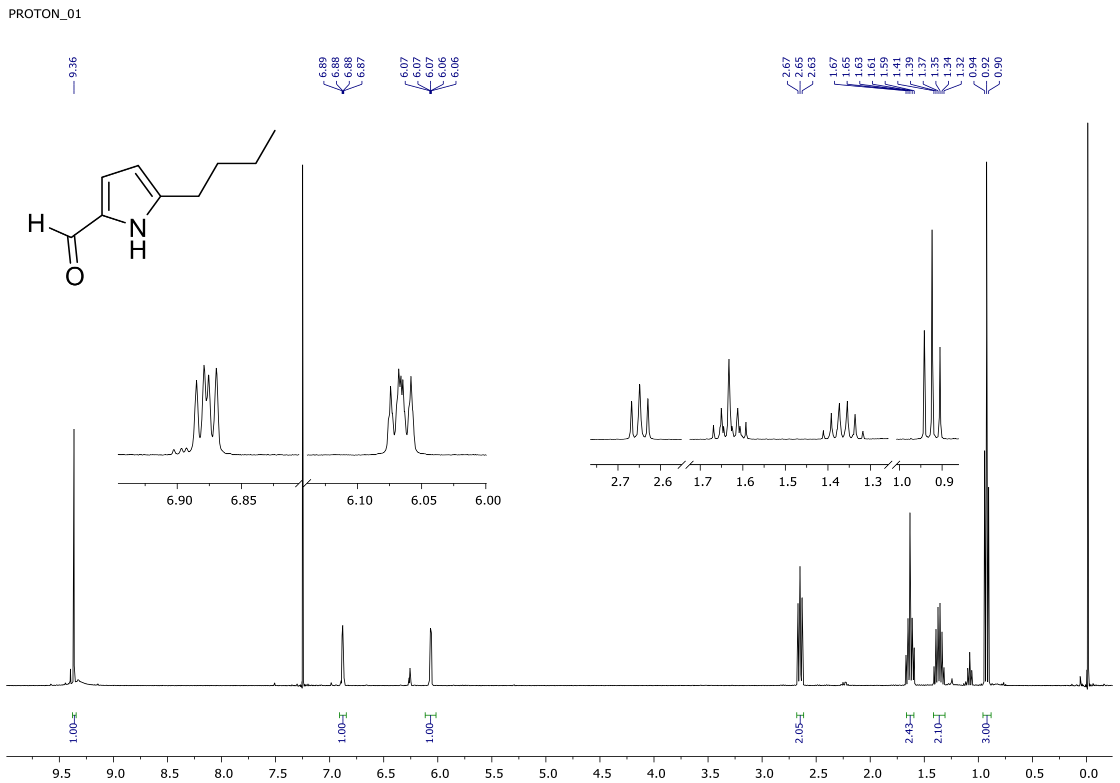


**Figure S22.** $^{1}$H-NMR of 2-Butyl-5-formylpyrrole.

**2-Ethyl-5-formylpyrrole**

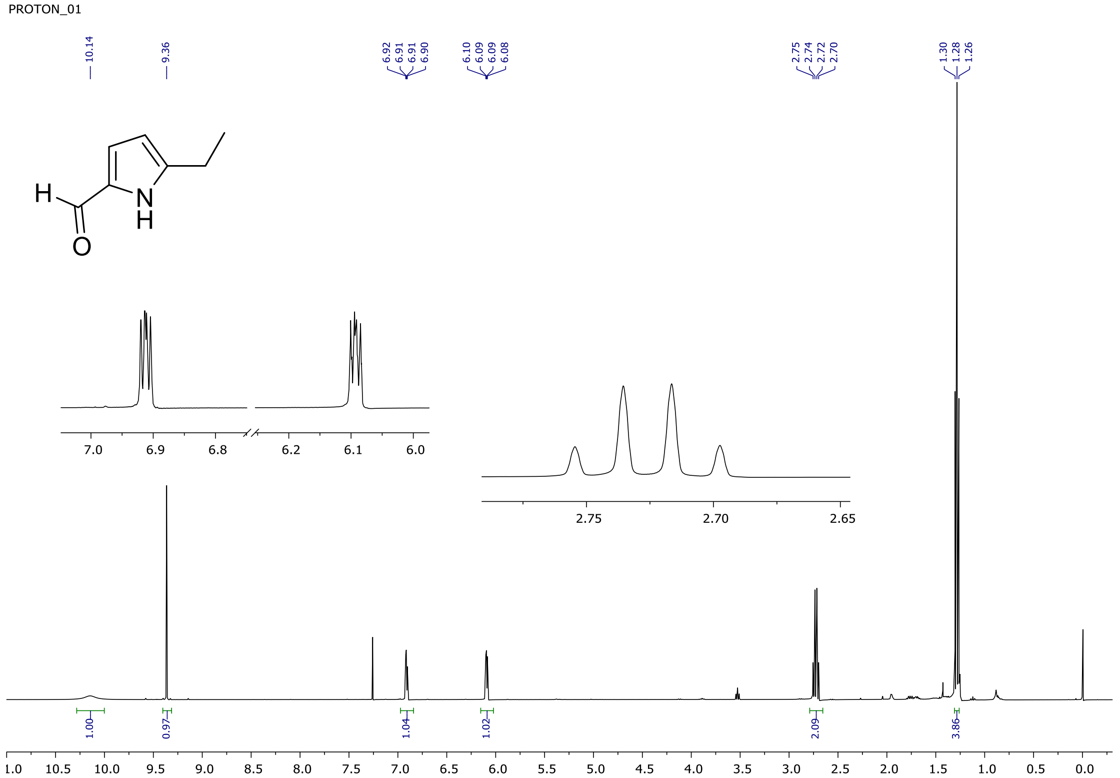


**Figure S23.** $^{1}$H-NMR of 2-Ethyl-5-formylpyrrole.

**2-Isopropyl-5-formylpyrrole**

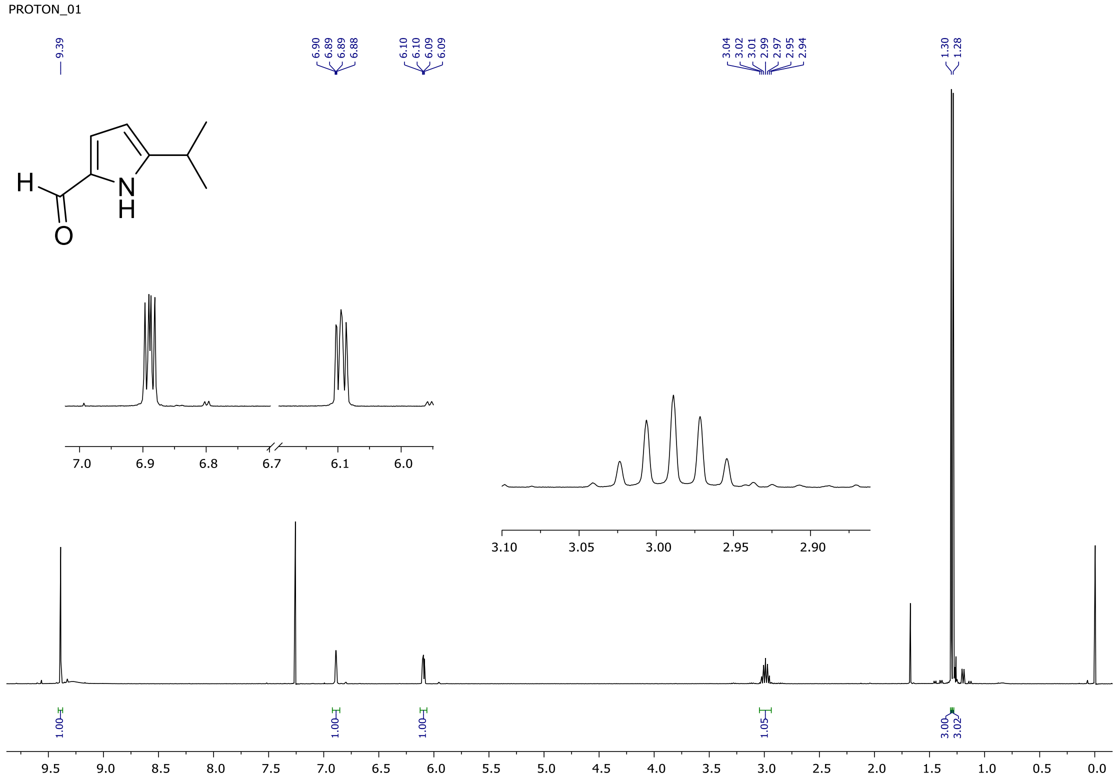


**Figure S24.** $^{1}$H-NMR of 2-Isopropyl-5-formylpyrrole.

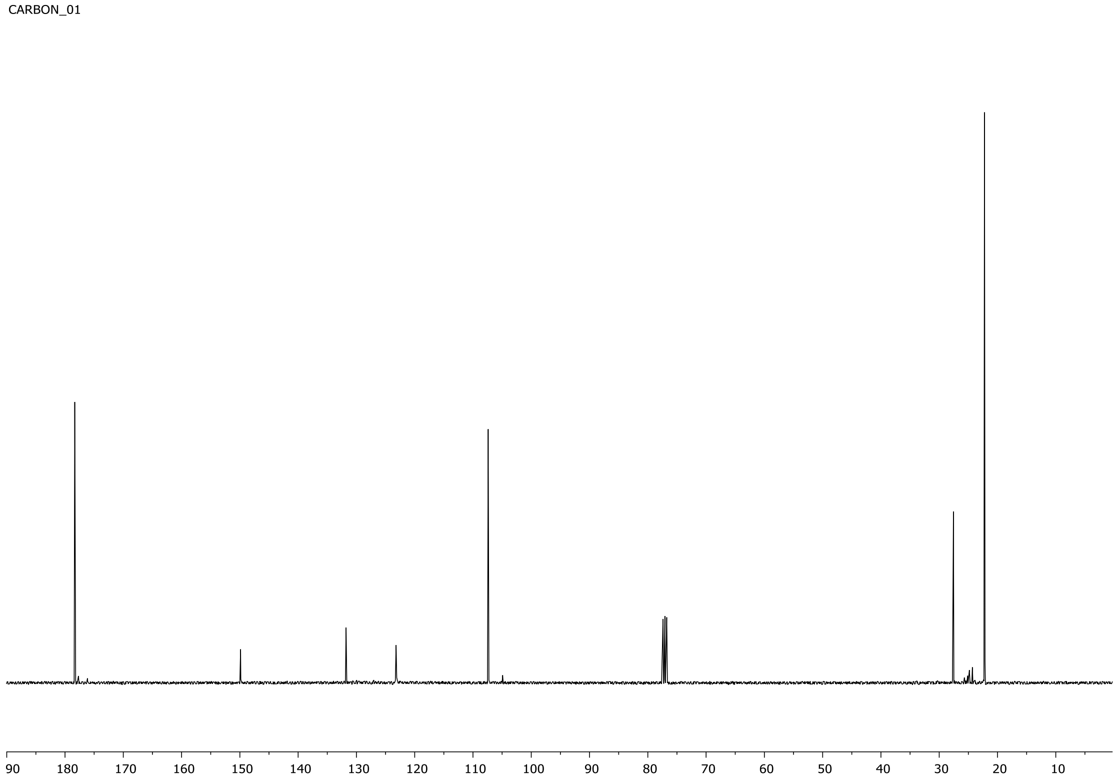


**Figure S25.** $^{13}$C-NMR of 2-Isopropyl-5-formylpyrrole.

**2-*sec*Butyl-5-formylpyrrole**

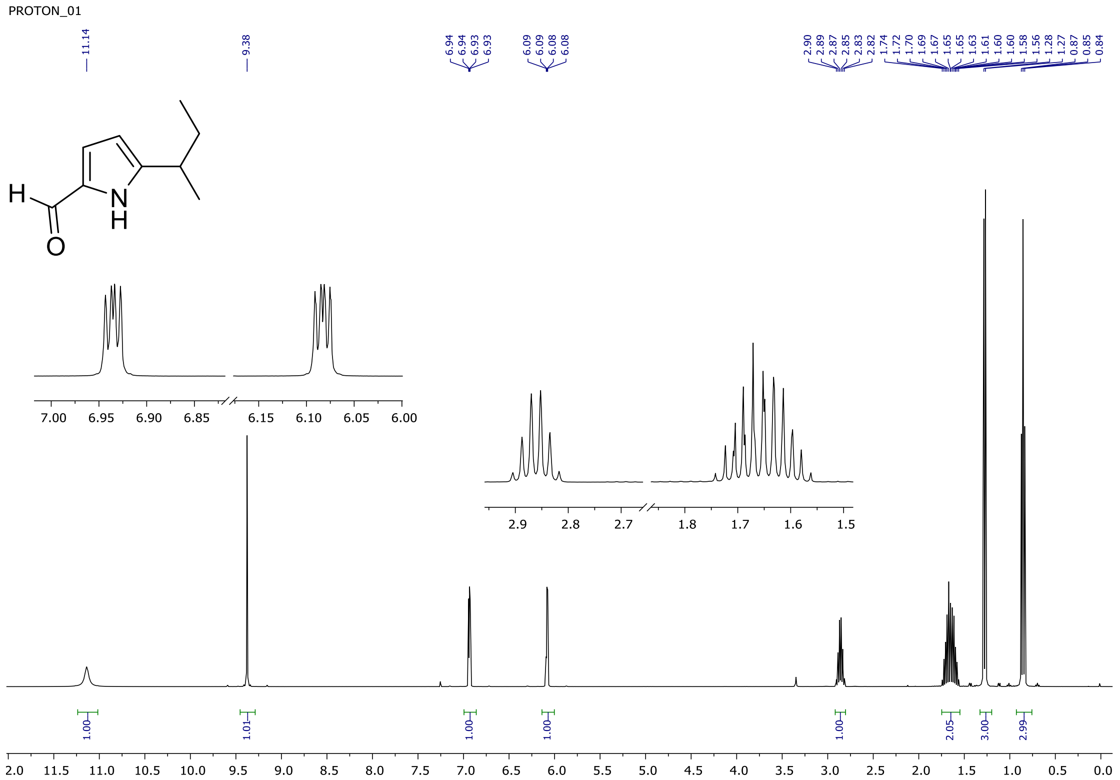

**Figure S26.** $^{1}$H-NMR of 2-*sec*butyl-5-formylpyrrole.

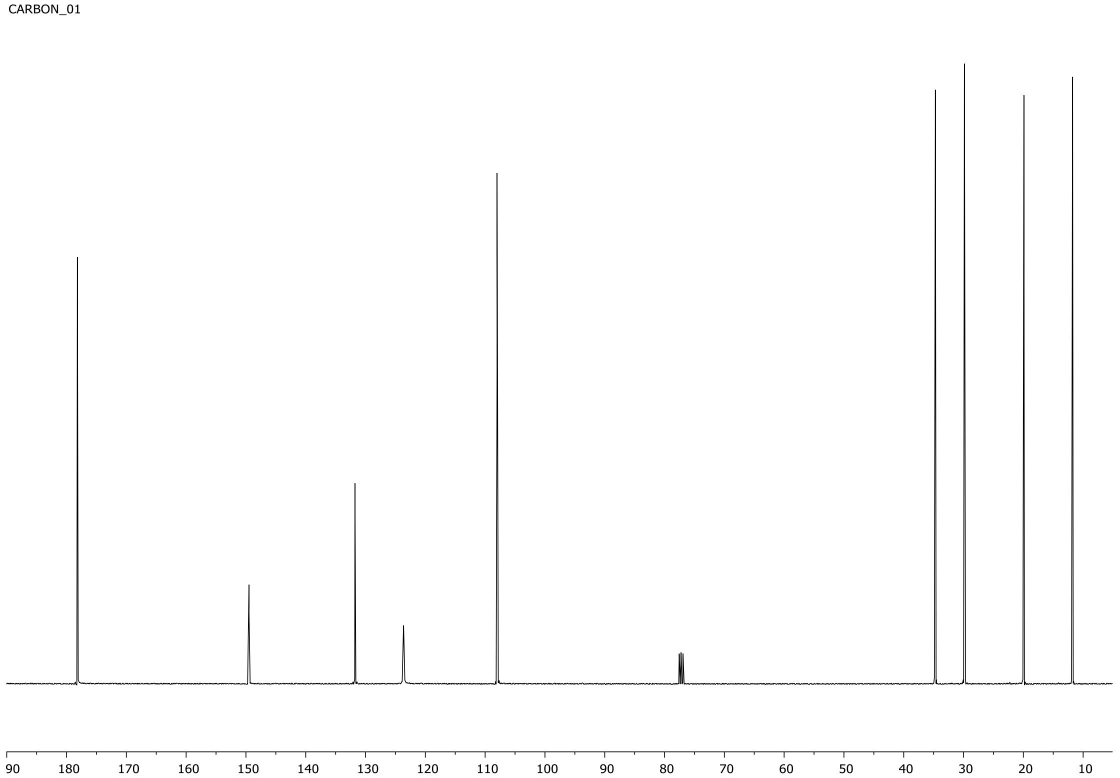

**Figure S27.** $^{13}$C-NMR of 2-*sec*butyl-5-formylpyrrole.

**2-*tert*Butyl-5-formylpyrrole**

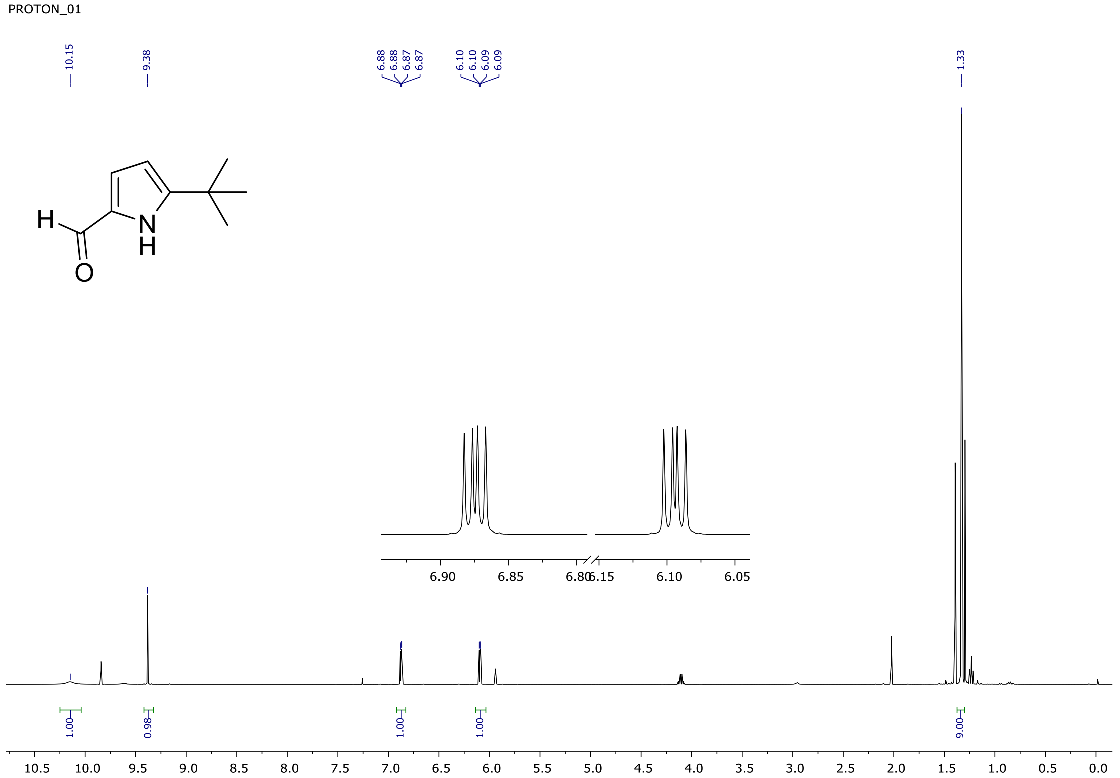


**Figure S28.** $^{1}H$-NMR of 2-*tert*butyl-5-formylpyrrole.

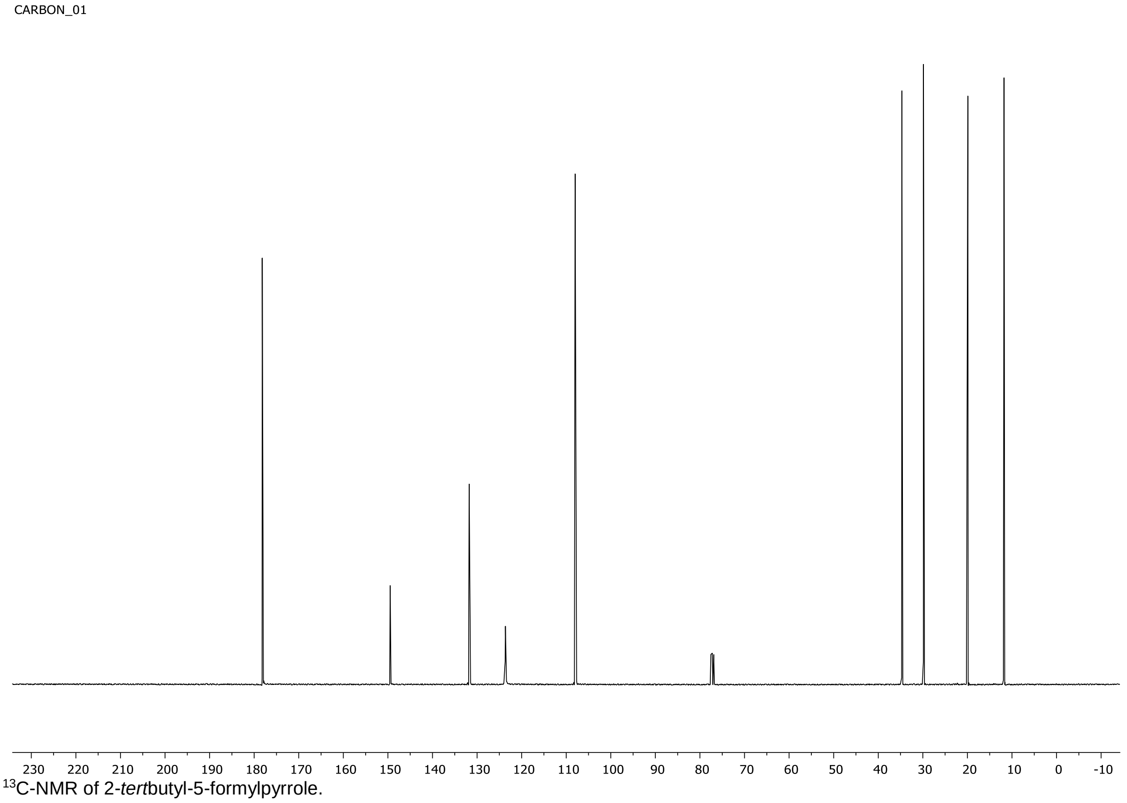


**Figure S29.** $^{13}C$-NMR of 2-*tert*butyl-5-formylpyrrole.

**α-EthylBODIPY**

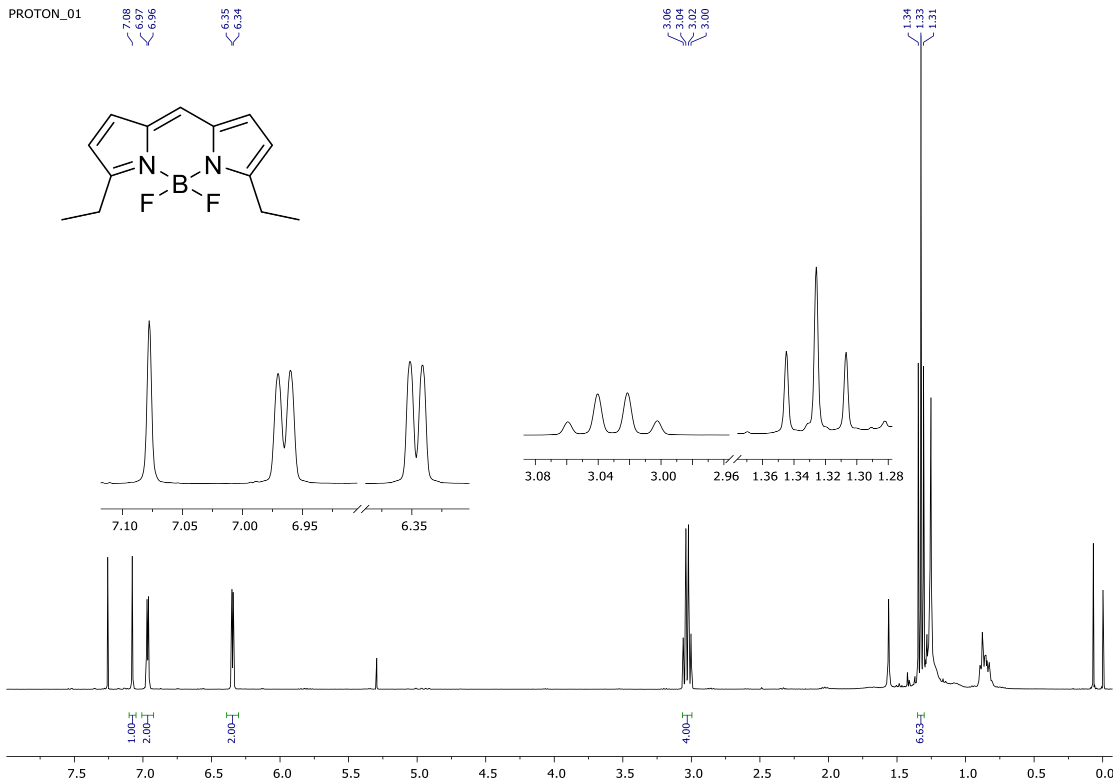


**Figure S30.** $^{1}$H-NMR of α-EthylBODIPY.

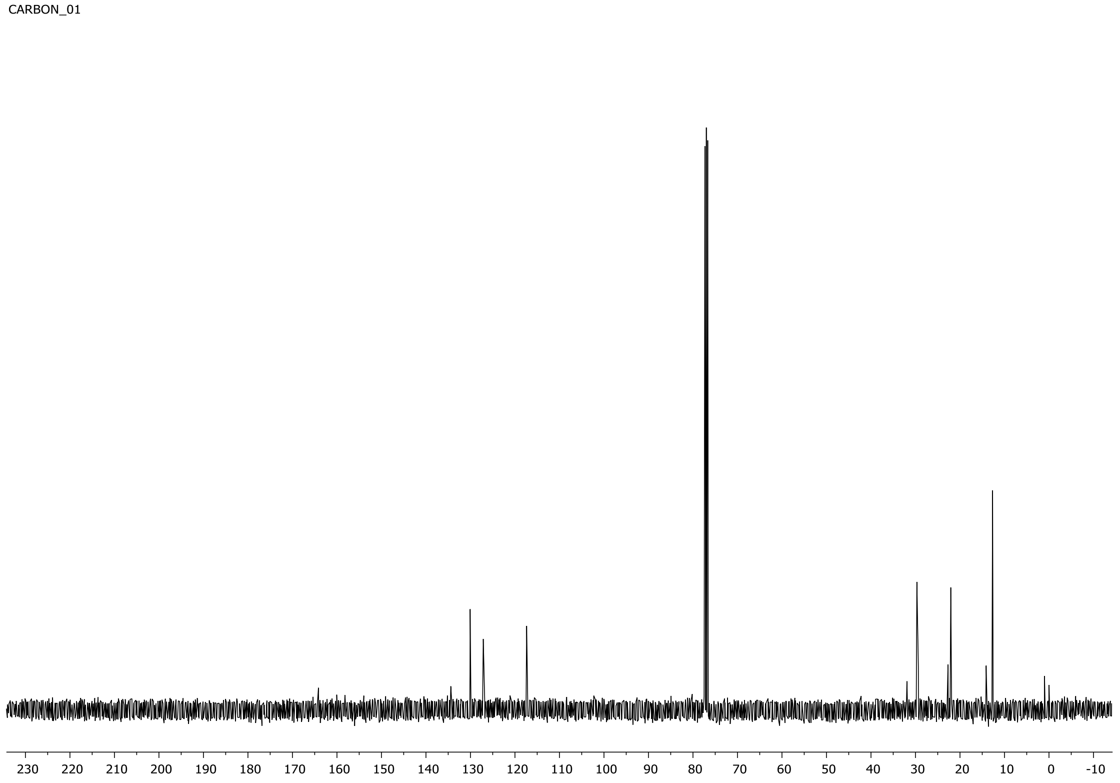


**Figure S31.** $^{13}$C-NMR of α-EthylBODIPY.

**α-*tert*ButylBODIPY**

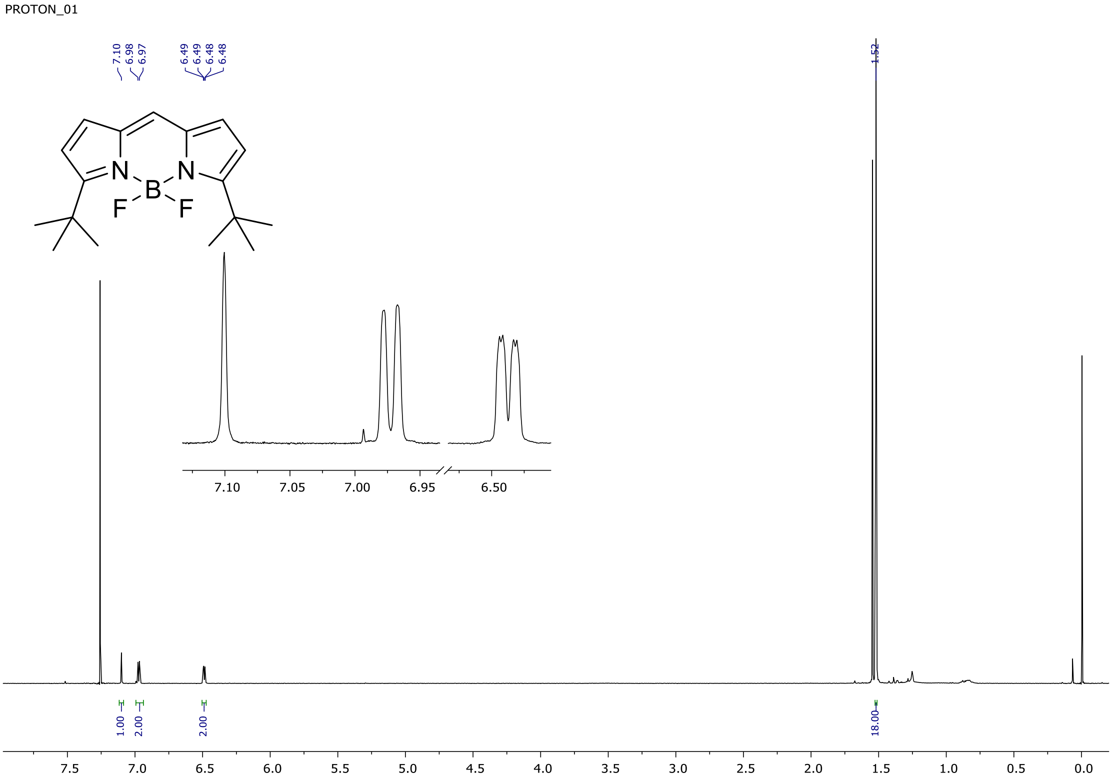


**Figure S32.** $^{1}$H-NMR of α-*tert*ButylBODIPY.

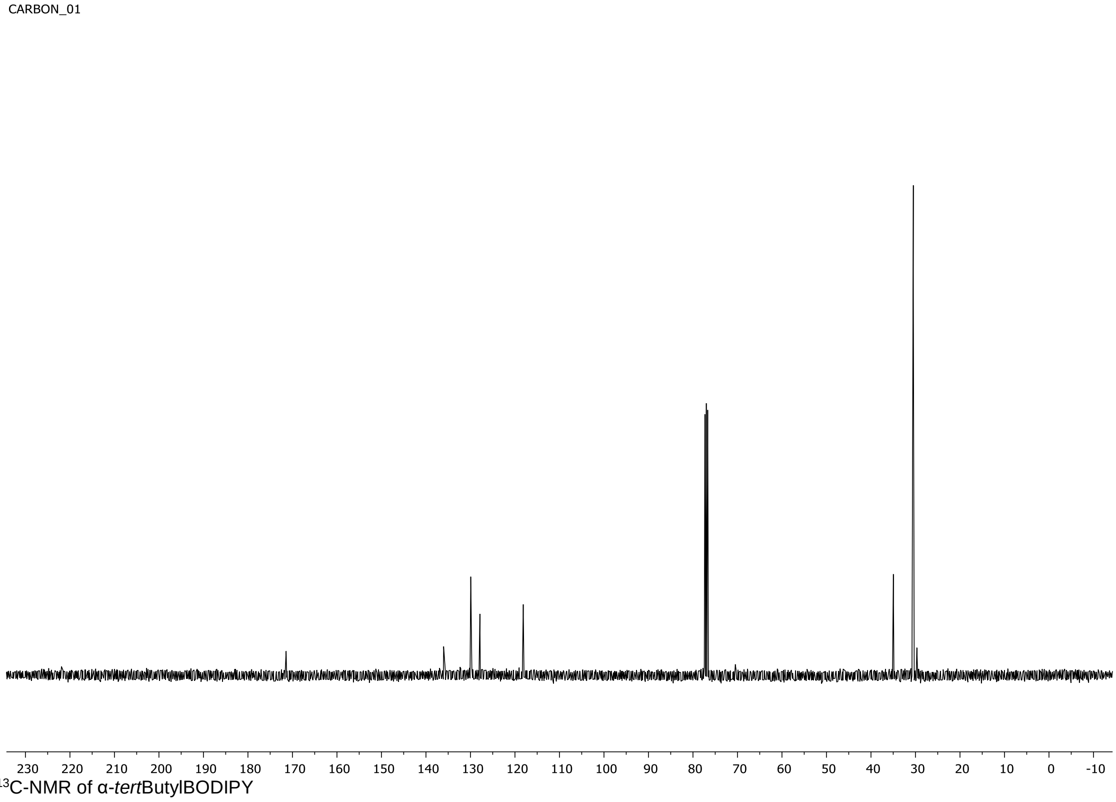


**Figure S33.** $^{13}$C-NMR of α-*tert*ButylBODIPY

**α-ButylBODIPY**

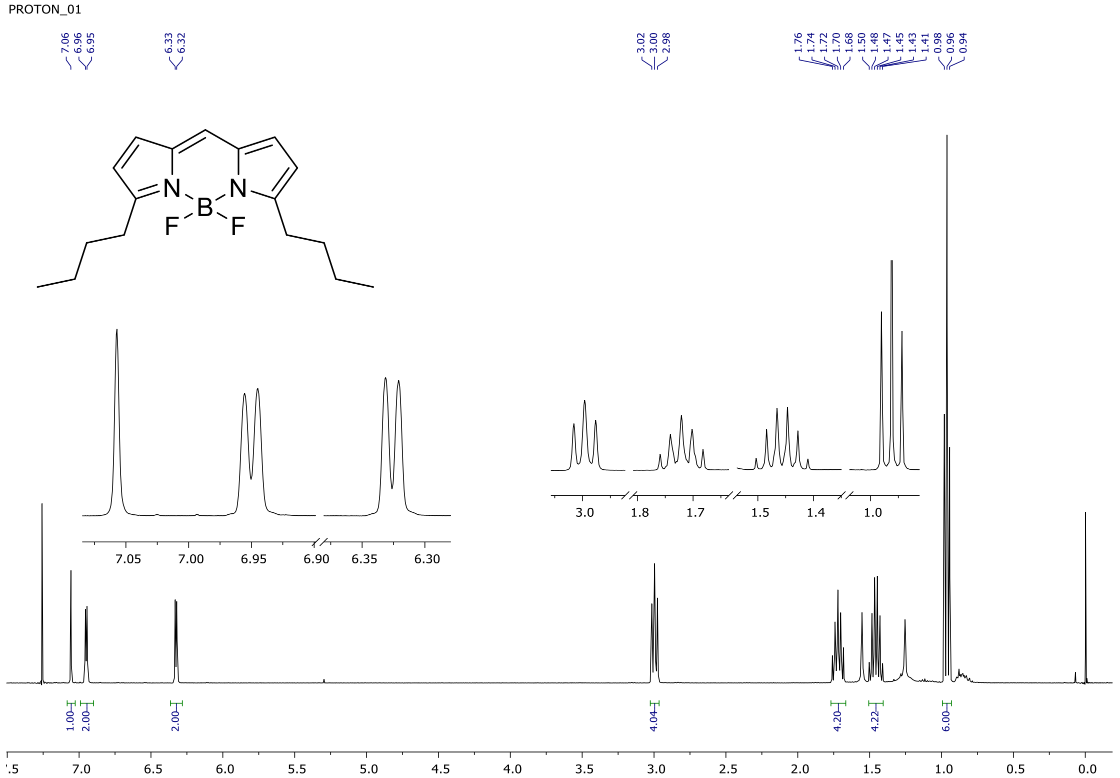

**Figure S34.** $^{1}$H-NMR of α-ButylBODIPY.

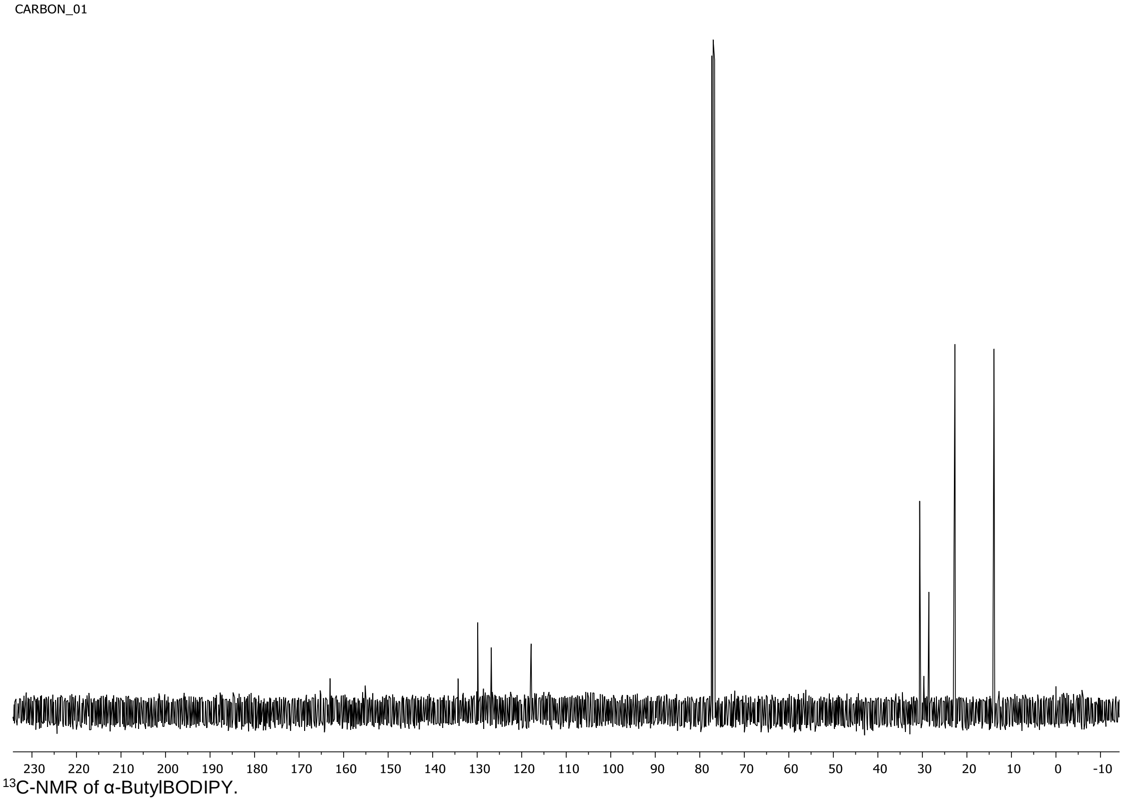

**Figure S35.** $^{13}$C-NMR of α-ButylBODIPY.

**α-*sec*ButylBODIPY**

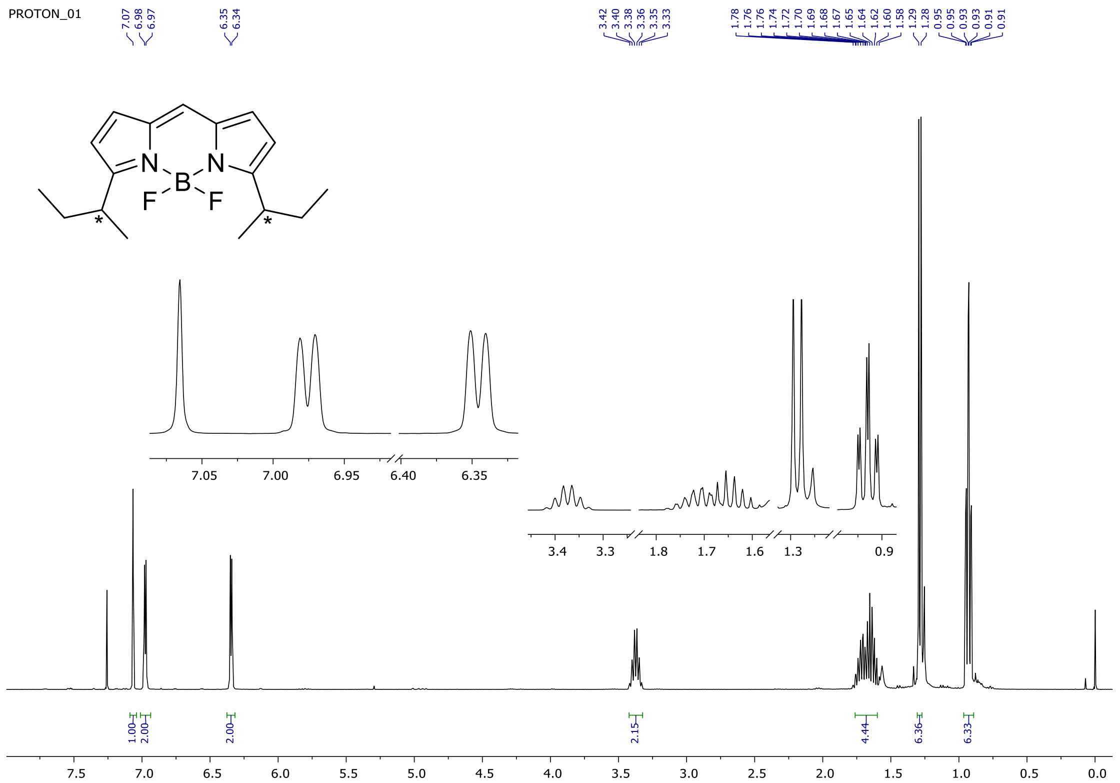


**Figure S36.** $^{1}$H-NMR of α-*sec*ButylBODIPY.

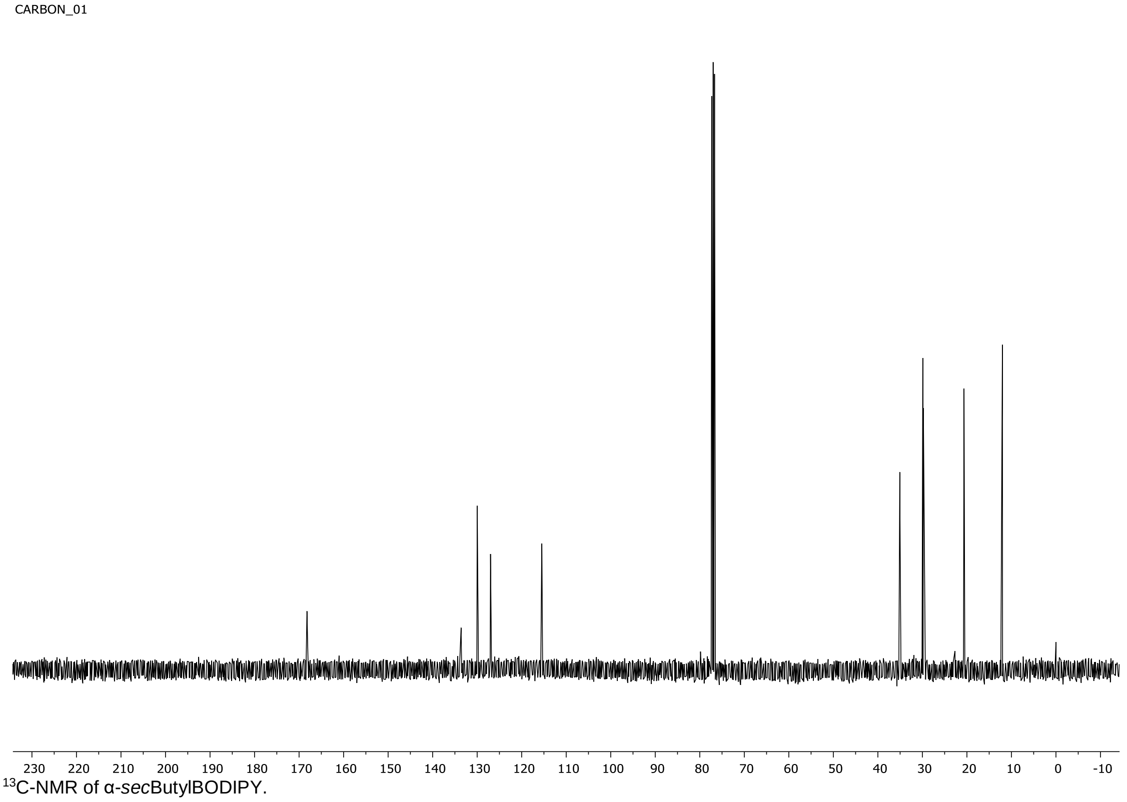


**Figure S37.** $^{13}$C-NMR of α-*sec*ButylBODIPY.

**α-IsopropylBODIPY**

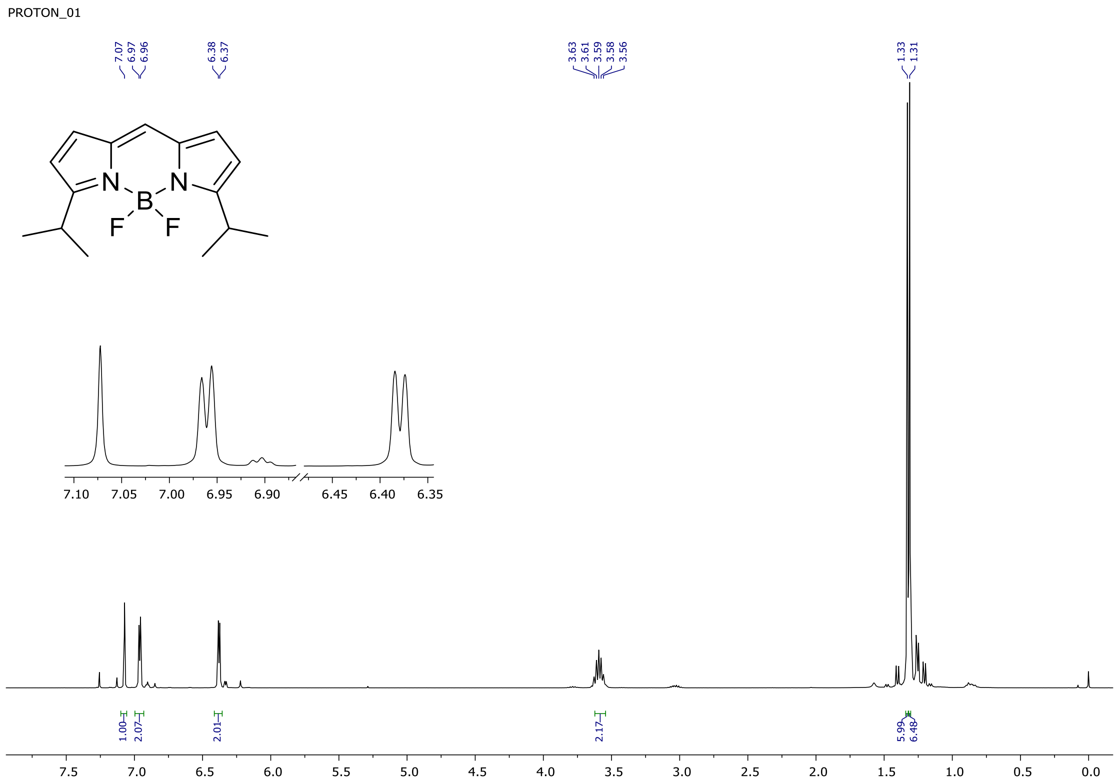


**Figure S38.** $^{1}$H-NMR of α-IsopropylBODIPY.

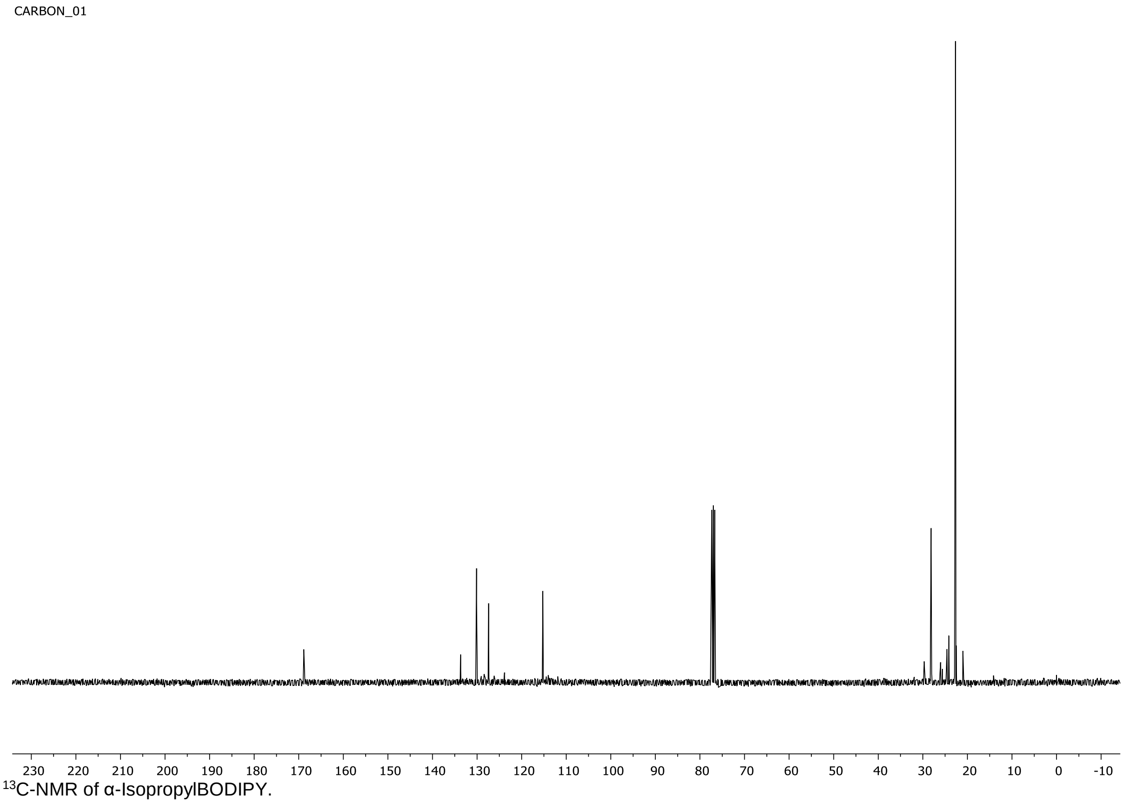


**Figure S39.** $^{13}$C-NMR of α-IsopropylBODIPY.

## References

[1] M. S. Skolnick, T. A. Fisher, D. M. Whittaker, *Semiconductor Science and Technology* **1998**, *13*, 645.
[2] P.-Q. Huang, Y.-H. Huang, K.-J. Xiao, *The Journal of Organic Chemistry* **2016**, *81*, 9020-9027.
[3] D. Znidar, A. O'Kearney-McMullan, R. Munday, C. Wiles, P. Poechlauer, C. Schmoelzer, D. Dallinger, C. O. Kappe, *Organic Process Research & Development* **2019**, *23*, 2445-2455.
[4] V. Tona, A. l. de la Torre, M. Padmanaban, S. Ruider, L. González, N. Maulide, *Journal of the American Chemical Society* **2016**, *138*, 8348-8351.
[5] R. G. Giles, H. Heaney, M. J. Plater, *Tetrahedron* **2015**, *71*, 7367-7385.
[6] E. Díez‐Barra, A. De la Hoz, A. Sánchez‐Migallón, A. Loupy, *Journal of Heterocyclic Chemistry* **1994**, *31*, 1715-1717.
[7] aP. Skell, G. Bean, *Journal of the American Chemical Society* **1962**, *84*, 4660-4661; bA. J. Castro, J. F. Deck, N. C. Ling, J. P. Marsh, G. E. Means, *The Journal of Organic Chemistry* **1965**, *30*, 344-350.
[8] aG. Broggini, C. La Rosa, T. Pilati, A. Terraneo, G. Zecchi, *Tetrahedron* **2001**, *57*, 8323-8332; bS. L. Berthiaume, B. L. Bray, P. Hess, Y. Liu, M. L. Maddox, J. M. Muchowski, M. E. Scheller, *Canadian journal of chemistry* **1995**, *73*, 675-684.
[9] L. Wu, K. Burgess, *Chem Commun (Camb)* **2008**, 4933-4935.
[10] L. M. Broomfield, J. A. Wright, M. Bochmann, *Dalton Transactions* **2009**, 8269-8279.
[11] L. Jiao, C. Yu, J. Wang, E. A. Briggs, N. A. Besley, D. Robinson, M. J. Ruedas-Rama, A. Orte, L. Crovetto, E. M. Talavera, *RSC Advances* **2015**, *5*, 89375-89388.

## Author Contributions

CS performed the synthesis and chemical characterization as well as the photophysical characterization of solutions and thin films and microscopic measurements of thin films; JM performed the photophysical analysis as well as preparation and analysis of the cavity; KB devised the conceptual idea and supervised the synthesis and photophysical characterization. TO contributed in supervising the synthesis. All authors contributed to writing the manuscript
